# Expression Patterns, Molecular Characterization, and Response to Host Stress of CYP Genes from *Phenacoccus solenopsis* (Hemiptera: Pseudococcidae)

**DOI:** 10.3390/insects10090264

**Published:** 2019-08-22

**Authors:** Lingyu Xi, Dan Liu, Lei Ma, Ying Zhang, Ruirui Sheng, Shaobing Zhang, Xiangli Dang, Guiting Li, Yong Miao, Junqi Jiang

**Affiliations:** Department of Entomology, College of Plant Protection, Anhui Agricultural University, Hefei 230036, China

**Keywords:** expression pattern, developmental stage, host plant, *P. solenopsis*, P450, quantitative real-time PCR

## Abstract

The quarantine insect pest *Phenacoccus solenopsis* (Hemiptera: Pseudococcidae) has a broad host range and is distributed worldwide. Each year, *P. solenopsis* causes significant crop losses. The detoxification of various xenobiotic compounds involves the cytochrome P450 monooxygenase (CYP) superfamily of enzymes. However, the functions of CYPs in *P. solenopsis* are poorly understood. In the present study, *P. solenopsis* was reared from the egg to the adult stage on three host plants: Tomato, cotton, and hibiscus. Thirty-seven *P. solenopsis* CYP genes were identified and their phylogenetic relationships were analyzed. Eleven CYP genes (*PsCYP4NT1*, *PsCYP4G219*, *PsCYP6PZ1*, *PsCYP6PZ5*, *PsCYP301B1*, *PsCYP302A1*, *PsCYP305A22*, *PsCYP315A1*, *PsCYP353F1*, *PsCYP3634A1*, and *PsCYP3635A2*) were selected for quantitative real-time PCR analysis. The results demonstrated marked differences in CYP expression levels in *P. solenopsis* grown on different host plants. The results will aid the molecular characterization of CYPs and will increase our understanding of CYP expression patterns in *P. solenopsis* during development and growth on different hosts.

## 1. Introduction

Generalist herbivores feed on a variety of plants and are therefore exposed to varied plant nutritional qualities and different secondary metabolites [1]. Plants produce certain defensive secondary substances, which are induced by insects feeding. These plant defensive secondary substances have certain advantages in plant defense against insects, because they can reduce the material and energetic costs of plant defense [2,3]. In the long-term process of evolution, generalist insects have developed mechanisms to cope with plant defenses, including behavioral avoidance against disease-resistant plants, the metabolising of toxic compounds, and even inhibition of induced defense by releasing inhibitors during feeding [4,5,6,7]. Host plants produce secondary metabolites that can induce the expression of related stress proteins, such as detoxifying enzymes, protective enzymes, digestive enzymes, and kinases, allowing the insects to adapt to the host plants to some extent [8].

Insects have evolved detoxification systems as a result of insect-plant interactions, which can accommodate plant secondary compounds via commonly found enzymes such as cytochrome P450 monooxygenases [9]. Cytochrome P450 enzymes can regulate the adaptability of several insect herbivores to host plants [10,11]. Cytochrome P450s can easily metabolize certain molecules that are harmful to the survival and reproduction of herbivores through their monooxygenase activity. P450 detoxification enzyme genes are one of the largest gene families in insects and are distributed in four CYP clans (CYP2, the mitochondrial clan, CYP3, and CYP4). Among them, CYP3 family members usually participate in the adaptability of herbivores to infect host plants [12]. The number of P450 genes in insects is highly variable; however, in general, the number of P450 genes in general omnivorous insects is much larger than that in oligophagous insects, possibly in response to different and unpredictable host challenges [13]. The induction of plant secondary substances upon the expression of cytochrome P450 genes in insects has also been widely reported [14,15,16,17]. Some plant secondary substances, such as benzoic acid and salicylic acid, which can inhibit the growth and development of insects, prolong the development period of insects, while others can also reduce insect reproduction [18]. The diversity of P450 species and the wide range of substrate specificities of P450 in organisms, mean that cytochrome P450s exert a variety of functions during the life cycle of insects. They are involved in the metabolism of pesticides and plant secondary substances, as well as the synthesis of ecdytin, juvenile hormone, and sex pheromones, which are closely related to insect growth, development, and defense [19].

*Phenacoccus solenopsis* Tinsley (Hemiptera: Pseudococcidae) is an economic pest, whose hosts are widespread around the world [20,21]. Since 2005, *P. solenopsis* has been recognized as posing a threat to ornamental plants, cotton, and vegetables. *P. solenopsis* affects in excess of 24 countries in Africa, Asia, Europe, and the Americas [22,23,24]. *P. solenopsis* secretes honeydew, which inhibits photosynthesis and produces sooty mold [25]. *P. solenopsis* can also be used as a vehicle to transmit plant diseases, such as hairy virus, cocoa bud virus, cotton leaf curl virus, and cocoa spotted leaf virus [26]. There have been many studies on the biology and ecology of *P. solenopsis* in its native and introduced ranges; however, no studies have been conducted on *P. solenopsis*’s cytochrome P450 gene expression after feeding on different hosts.

In the present study, the transcriptomes of *P. solenopsis* grown on three host plants: Tomato, cotton, and hibiscus were determined. In the transcriptome data, 37 CYP genes were identified and their phylogenetic relationships were analyzed. We selected 11 CYP genes (*PsCYP4NT1*, *PsCYP4G219*, *PsCYP6PZ1*, *PsCYP6PZ5*, *PsCYP301B1*, *PsCYP302A1*, *PsCYP305A22*, *PsCYP315A1*, *PsCYP353F1*, *PsCYP3634A1*, and *PsCYP3635A2*) for quantitative real-time PCR analysis. The results showed that when *P. solenopsis* was grown on different host plants, significant differences in CYP gene expression could be observed. The results provided a theoretical basis for future research on *P. solenopsis*.

## 2. Materials and Methods

### 2.1. Host Plants and Insects

Samples of *P. solenopsis* were collected from cotton in Guangdong Province in May 2003. The experimental tomato population was grown at 26 °C and 75% relative humidity with 14 h of light and 10 h of dark in an artificial climate chamber.

*P. solenopsis* were raised for many generations on three host plants: Tomato (Shanghai 906), hibiscus, and cotton (China’s Hebei cotton 169). Cotton and tomato were grown artificially in greenhouses, and hibiscus was grown hydroponically.

### 2.2. Extraction of RNA and Preparation of RNA-Seq Libraries

The Trizol reagent (Invitrogen, Carlsbad, CA, USA) was used to extract total RNA from *P. solenopsis* at different developmental stages, according to the manufacturer’s procedure. A Bioanalyzer 2100 and RNA 6000 Nano LabChip Kit (Agilent, Santa, Clara, CA, USA) were used to assess the amount and purity of the total RNA, with an RNA integrity value (RIN) value > 7.0 being considered acceptable. RNAs with poly(A) sequences were isolated from about 10 µg of total RNA representing different developmental stages and hosts using poly-T oligomerized magnetic beads (Invitrogen). After purification, high temperature and polyvalent cations were used to segment the poly(A)− or poly(A)+ RNA into small pieces. Reverse transcription of the RNA fragments was used to generate the final cDNA library using Illumina technology (San Diego, CA, USA) based on the RNA-Seq sample. The paired-end library had an average insertion fragment size of 300 bp (±50 bp). The paired-end cDNA library was sequenced using an Illumina Hiseq 4000 sequencer (LC-bio, Hangzhou, China) according to the supplier’s protocol.

### 2.3. De Novo Assembly of Unigenes Annotation, and Functional Classification

First, internally developed Perl scripts and Cutadapt [27] were used to remove reads containing linker contamination, undetermined bases, and low-quality bases. FastQC (http://www.bioinformatics.babraham.ac.uk/projects/fastqc/) was then used to validate the sequence quality, including the Q20 and Q30 values, and the GC content of the clean data. All downstream analysis was based on the high-quality clean data. Trinity 2.4.0 [28] was used for de novo assembly of the transcriptomes, in which shared sequence content was used to group transcripts into clusters. These clusters of transcripts were referred to as “genes.” In each cluster, the longest transcript was chosen as the representative “gene” sequence, and termed a unigene.

DIAMOND [29] was used to align all the assembled unigenes with the non-redundant (Nr) protein databases (http://www.ncbi.nlm.nih.gov/), SwissProt (http://www.expasy.ch/sprot/), Gene ontology (GO) (http://www.geneontology.org), the Kyoto Encyclopedia of Genes and Genomes (KEGG) (http://www.genome.jp/kegg/), and eggNOG (evolutionary genealogy of genes: Non-supervised Orthologous Groups; http://eggnogdb.embl.de/) databases, using a threshold E-value of less than 0.00001.

### 2.4. Bioinformatics Analyses

We predicted signal peptides, the isoelectric point, and conserved domains using SignalP (http://www.cbs.dtu.dk/services/SignalP/), Compute pI (https://web.expasy.org/compute_pi/), and SMART (http://smart.embl-heidelberg.de/), respectively. The MEGA 7.0 software (Tempe, AZ, USA) [30] was used to construct a phylogenetic tree, utilizing the neighbor-joining method with 1000 bootstrap replications. Finally, we submitted each *P. solenopsis* CYP gene sequence to the cytochrome P450 nomenclature committee (D. Nelson, University of Tennessee, Memphis, TN, USA).

### 2.5. Quantitative Real-Time PCR

We selected 11 CYP genes that seemed to be involved in detoxification (Table 1) and used Primer Premier 5 to design primers for quantitative real-time PCR (qPCR). The housekeeping gene, *P. solenopsis* α-tubulin (GenBank accession no. KJ909508), was used as the endogenous control.

A 2× Plus SYBR real-time PCR mixture (BioTake, Beijing, China) was used to perform the qPCR reactions. The reactions comprised 10 μL of 2× Plus SYBR real-time PCR mixture, 1 μL (10 ng) of cDNA template, 0.5 μL of sense primer, 0.5 μL of anti-sense primer (0.2 μM), and 8.0 μL diethyl pyrocarbonate (DEPC)-ddH_2_O. The reactions were run on a Bio-Rad CFX96 system (Bio-Rad, Hercules, CA, USA) using an amplification protocol as follows: 94 °C for 60 s, followed by 39 cycles of 94 °C for 15 s and 60 °C for 30 s. Each experiment was run in three biological replicates, and relative expression levels of P450 genes across various samples were determined the 2^−^^ΔΔCt^ method. The DPSv7.5 [31] software was to analyze the qPCR results of *P. solenopsis* in different hosts and at different ages. A *p*-value < 0.05 was regarded as indicating statistical significance.

## 3. Results

### 3.1. Assembly and Annotation of Unigenes

**Figure 1:**
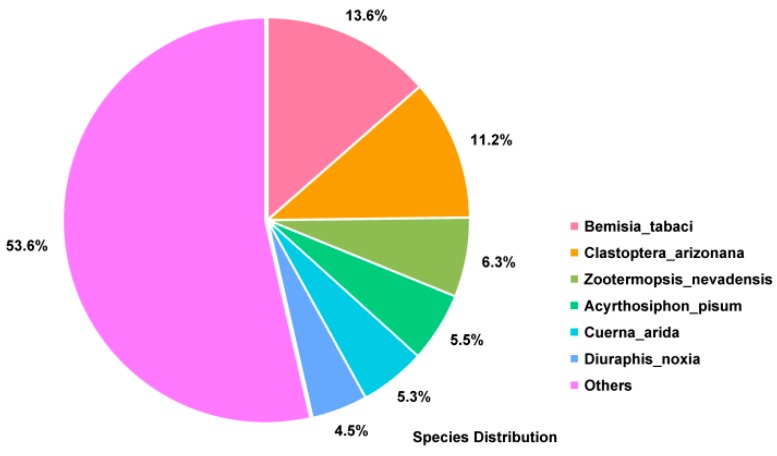
The *P. solenopsis* transcriptome profile. The pie chart shows the similarity between the transcriptome data of other species deposited in the non-redundant (NR) database and that of *P. solenopsis*.

Sequencing of the *P. solenopsis* transcriptome produced 47,635,589 clean reads. Assembly of the clean reads resulted in 35,352 unigenes with an accumulated length of 30,633,522 bp. The longest gene was 13,825 bp and the N50 value was 1680 bp (Table S1). To compare the obtained unigene sequences with the protein sequences in public databases (SwissProt, NR, KEGG, KOG, and Pfam), BLASTX searching (threshold E ≤ 0.00001) was performed. The results of the searches annotated 10,271 genes in the Pfam database, (29.05% of the total number of unigenes); 9977 in the GO database (28.22%); 11,886 in the eggNOG database (33.62%), and 6883 in the KEGG database (19.47%). Among the unigenes, 12,373 (53.57%) sequences matched sequences from other species (Table S2). The highest number of matches was between *P. solenopsis* unigenes and sequences from *Bemisia tabaci* (13.6%) (Figure 1).

### 3.2. GO, KEGG, and eggNOG Classification

**Figure 2:**
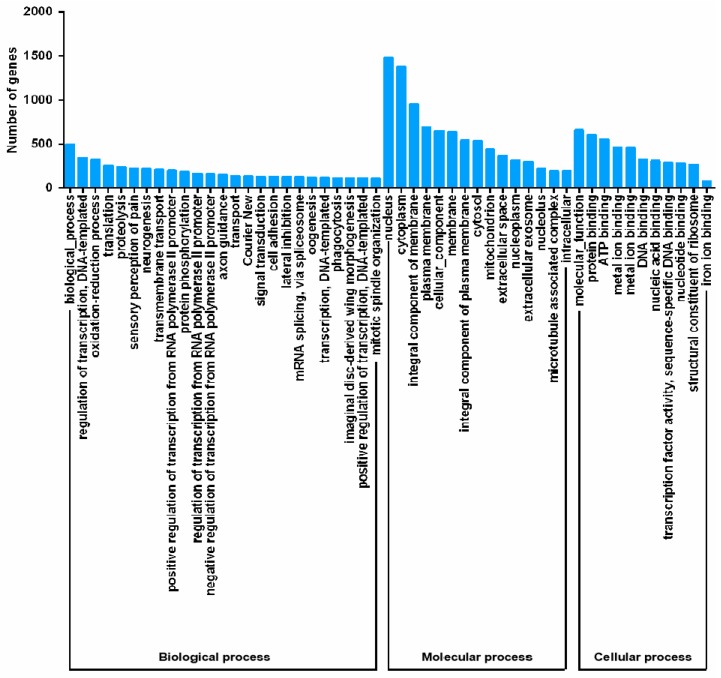
Gene ontology (GO) classification of unigenes.

To better classify the functions of *P. solenopsis* unigenes, GO analysis was carried out (Figure 2). The results showed that 9977 of the 35,352 unigenes (28.22%) corresponded to at least one GO term. Various GO terms from the three domains (“biological process,” “cellular component,” and “molecular function”) could be assigned to the *P. solenopsis* transcripts. Among the 50 GO categories, “nucleus” (1481 unigenes), “cytoplasm” (1372 unigenes), and “integral component of membrane” (957 unigenes) were the most dominant molecular functions.

**Figure 3:**
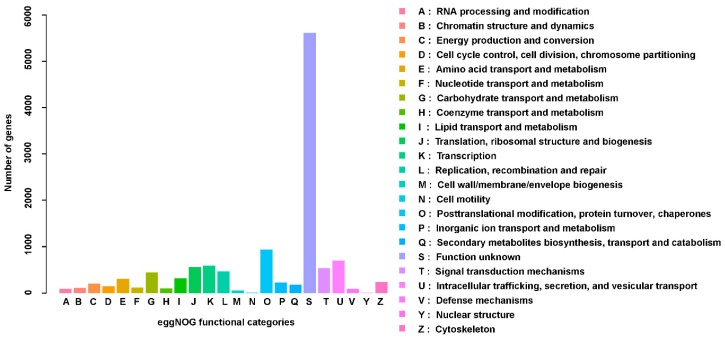
Non-supervised orthologous groups analysis showing the evolutionary genealogy of the genes.

The results of the eggNOG analysis showed that among the 35,352 tested *P. solenopsis* unigenes, 11,886 (33.62%) could be classified using eggNOG (Figure 3). In eggNOG, matches to the cluster “function unknown” represented the largest group, followed by the “posttranslational modification, protein turnover, chaperones” and “intracellular trafficking, secretion, and vesicular transport”.

**Figure 4:**
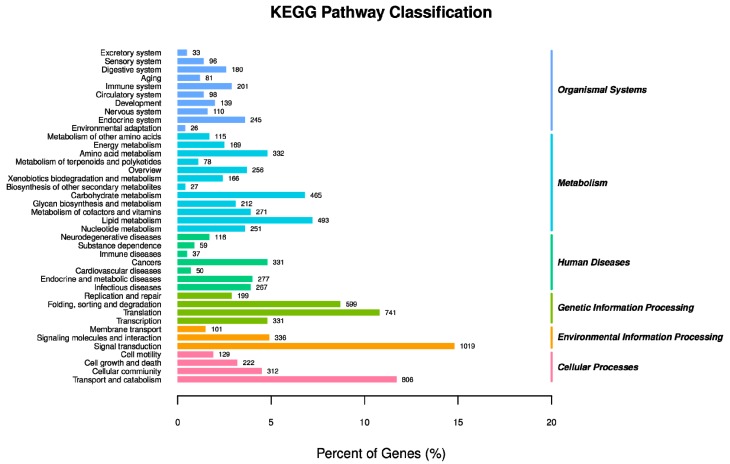
Classification of *P. solenopsis* unigenes using the Kyoto Encyclopedia of Genes and Genomes.

KEGG pathways were matched by 6883 unigenes. The largest number of contigs could be annotated as environmental information processing of “signal transduction” (1019 unigenes), followed by “transport and catabolism” (806 unigenes) in cellular processes, “translation” (741 unigenes) and “folding, sorting and degradation” (599 unigenes) in genetic information processing (Figure 4).

### 3.3. Identification of Cytochrome P450 Monooxygenases Genes in P. solenopsis

In *P. solenopsis*, a total of 37 CYP genes were identified among the unigenes (Table 2). Among them, 28 had full open reading frames (ORFs), and ninr genes were incomplete, with truncated 5′ and/or 3′ coding regions. The intact ORFs of the CYPs ranged from 435 to 577 amino acids. Using the accepted CYP nomenclature, the 37 CYP sequences were divided into 17 families and 20 subfamilies, of which CYP6 was the largest family, with 17 genes. The next largest family was CYP4 with seven members. BLASTX searching using the deduced protein sequences indicated that the amino acid identities between these CYPs and their hemipteran orthologs ranged from 33% to 79% (Table S3).

### 3.4. P. solenopsis P450s Sequence Analysis

Sequence analysis of the encoded proteins of 11 CYP genes (*PsCYP4NT1*, *PsCYP4G219*, *PsCYP6PZ1*, *PsCYP6PZ5*, *PsCYP301B1*, *PsCYP302A1*, *PsCYP305A22*, *PsCYP315A1***,** *PsCYP353F1*, *PsCYP3634A1*, and *PsCYP3635A2*) showed that none were predicted to have signal peptides or transmembrane domains. The isoelectric points of the eleven CYPs were predicted and are shown in Table 3.

All 11 P450 amino acid sequences from *P. solenopsis* contain a characteristic cysteine heme-iron ligand domain, and the PsCYP302A1 amino acid sequence contains a hemopexin domain signature (Figure S1).

### 3.5. Phylogenetic Analysis of P450s from P. solenopsis

**Figure 5:**
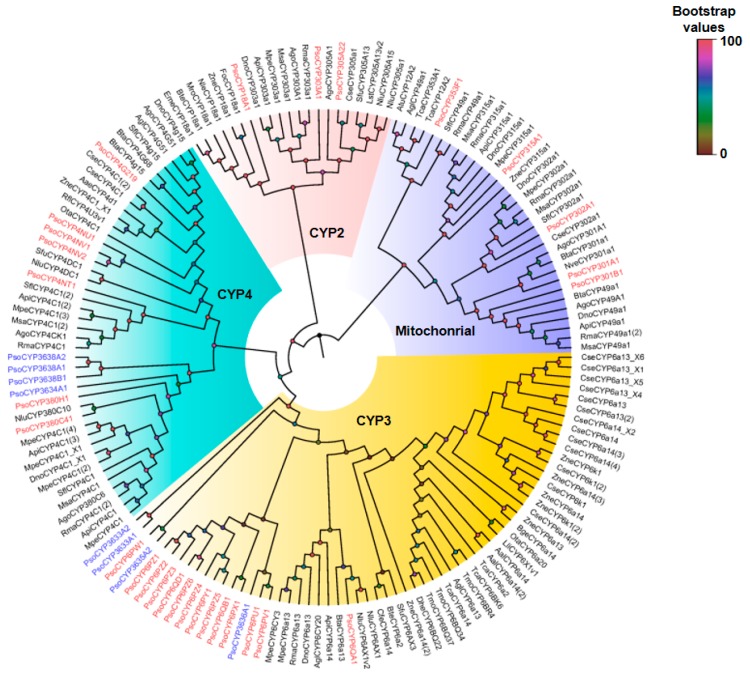
A phylogenetic tree of *P. solenopsis* CYPs with CYPs from other species constructed using the neighbor-joining method in MEGA software (version: 7.0). Each branch shows Bootstrap values from 1000 replications. *P. solenopsis* CYPs are shown in red and *P. solenopsis* new family CYPs are shown in blue. Table S4 lists the CYP sequences used in this analysis.

A phylogenetic tree was constructed using the neighbor-joining method to analyze the relationships among the 37 CYP proteins from *P. solenopsis* and those from other species of insect (Figure 5). The phylogenetic tree showed that insect CYPs could be classified into four categories (clans): Mitochondrial, CYP2, CYP3, and CYP4. Among them, CYP3 and CYP4 clans accounted for the majority of genes, while the mitochondrial and CYP2 contained only nine proteins. Clan CYP2 includes three families; namely, CYP18A1, CYP303A1, and CYP305A22. Certain *P. solenopsis* CYPs were found to be homologous to members of the mitochondrial family, including PsCYP301A1, PsCYP301B1, PsCYP302A1, PsCYP315A1, and PsCYP353F1. Gene amplification was observed to have occurred in the CYP4 and CYP3 clans. For example, the largest cluster in the CYP3 clan was CYP6, whereas CYP4 genes (plus one CYP380 cluster) formed the largest cluster in the CYP4 clan. In addition, we found five new families of P450s: PsCYP3633A1 and PsCYP3633A2; PsCYP3634A1; PsCYP3635A2; PsCYP3636A1; and PsCYP3638A1, PsCYP3638A2, and PsCYP3638B1. Interestingly, PsCYP3634A1, PsCYP368A1, PsCYP3638A2, PsCYP3638B1, and the CYP4 clan clustered together; whereas, PsCYP3633A1, PsCYP33A2, PsCYP3636A1, PsCYP3635A2, and CYP6 clan clustered together.

### 3.6. Expression of P450s after P. solenopsis Feeding on Three Host Plants

**Figure 6:**
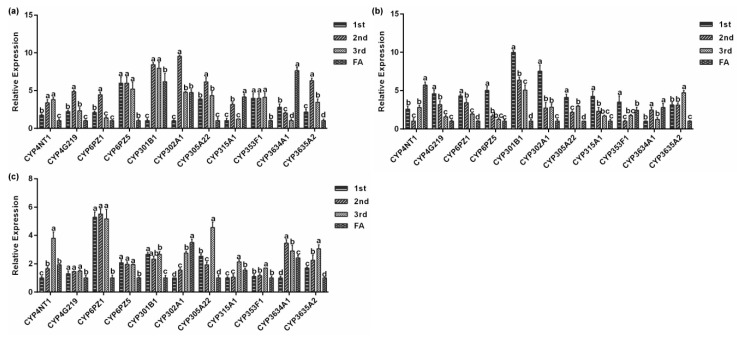
Relative expression levels of *P. solenopsis* CYP genes in different developmental stages. (**a**) tomato, (**b**) cotton, and (**c**) hibiscus. Data are presented as the mean of three replicates (*n* = 3) ± standard error. Different lowercase letters indicated that there were significant differences in CYP gene expression among different developmental stages of *P. solenopsis* fed on the same host.

**Figure 7:**
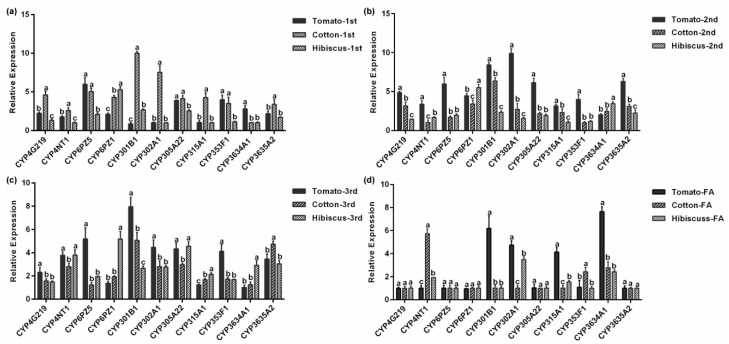
Relative CYP gene expression in *P. solenopsis* on different hosts. (**a**) The first instar nymph. (**b**) The second instar nymph. (**c**) The third instar nymph. (**d**) The female adult. Data show the mean ± standard error (*n* = 3). Different lowercase letters indicate significant differences in CYP expression among different hosts of *P. solenopsis* at the same developmental stage.

Next, we assessed the relative mRNA levels of the eleven *P. solenopsis* CYP genes at different stages of development when fed on three host plants. The 11 CYP genes showed differential expression at different developmental stages of *P. solenopsis* (Figure 6a–c) and the expression levels at the same developmental stage were also different in different hosts (Figure 7a–d). After feeding on tomato, the expression of *PsCYP301B1* was the lowest in the first instar nymph and *PsCYP6PZ5* was the highest by a factor of 7.0 (*p* < 0.05, Figure 6a) compared with that of *PsCYP301B1*. In the second instar nymph, *PsCYP302A1* had the highest expression level, which was 4.9 times (*p* < 0.05, Figure 6a) higher than that of *PsCYP3634A1*, which showed the lowest expression level. The expression level of *PsCYP3634A1* was the lowest in the third instar nymph, while the expression level of *PsCYP301B1* was the highest, showing 7.9 times (*p* < 0.05, Figure 6a) higher expression than that of *PsCYP3634A1*. In the female adult, *PsCYP3634A1* had the highest expression level, which was 7.6 times (*p* < 0.05, Figure 6a) higher than the lowest level of *PsCYP4G219* expression.

The expression of *PsCYP301B1* in first instar nymphs was significantly higher than that of other genes when fed on cotton, being was 10.0 times (*p* < 0.05, Figure 6b) the lowest expression of *PsCYP3634A1*. The expression level of *PsCYP353F1* was the lowest in the second instar nymphs, while the highest expression level of *PsCYP301B1* was 6.3 times higher (*p* < 0.05, Figure 6b) than that of *PsCYP353F1*. *PsCYP3635A2* showed its highest expression level in the third instar nymphs, at 4.6 times (*p* < 0.05, Figure 6b) the expression of *CYP6PZ5*, which showed the lowest expression level. In the female adult, *PsCYP4NT1* had the highest expression level, which was 5.7 times (*p* < 0.05, Figure 6b) higher than that of *PsCYP305A22*, which showed the lowest level of expression.

When the insects were fed on hibiscus, *PsCYP6PZ1* had the highest expression level in the first instar nymph, the second instar nymph, and the third instar nymph. In the first instar nymph, the expression level of *PsCYP6PZ1* was 5.3 times (*p* < 0.05, Figure 6c) higher than that of *PsCYP302A1*. In the second instar nymph, the expression level of *PsCYP6PZ1* was 5.2 times (*p* < 0.05, Figure 6c) higher than that of *PsCYP315A1*. The expression level of *PsCYP6PZ1* was 3.5 times (*p* < 0.05, Figure 6c) higher than that of *PsCYP4G219* in the third instar nymph. The expression level of *PsCYP3635A2* was the lowest in the third instar nymph, while the highest expression level of *PsCYP302A1* was 3.5 times (*p* < 0.05, Figure 6c) higher than that of *PsCYP3635A2*.

Figure 7a–d shows the expression levels of the 11 *P. solenopsis* CYP genes at the same developmental stage after feeding on three host plants. In first instar nymph feeding on cotton, *PsCYP301B1* and *PsCYP302A1* expression levels were significantly higher compared with those of other CYP genes, while the expression levels of *PsCYP302A1* were the lowest after the insects were fed on hibiscus (*p* < 0.05, Figure 7a). Among the second instar nymphs, *PsCYP302A1* showed its highest expression when fed on tomatoes, which was 9.6 times (*p* < 0.05, Figure 7b) higher than that of *PsCYP4NT1*, which showed the lowest expression on cotton. The expression of *PsCYP301B1* was the highest in the third instar nymph. However, in the third instar nymphs, the expression of *PsCYP3634A1* was significantly lower after tomato feeding compared with that of the other CYP genes, and the expression of *CYP301B1* was 7.9 times (*p* < 0.05, Figure 7c) higher than that of *PsCYP3634A1*. In the female adult stage, we observed no significant difference in the expression levels of *PsCYP4G219*, *PsCYP6PZ1*, *PsCYP6PZ5*, *PsCYP305A22*, and *PsCYP3635A2* after feeding on the three hosts. However, the expression levels of *PsCYP3634A1* after feeding on tomatoes were the highest by 8.0 times (*p* < 0.05, Figure 7d) compared with that of *PsCYP6PZ1*.

## 4. Discussion

The present study revealed the expression patterns of *P. solenopsis* CYP genes after feeding on three different hosts. Generalist herbivores usually feed on a variety of plants; therefore, they need to adapt to different host qualities and defenses. In the course of these long-term interactions, omnivorous insects have evolved mechanisms to overcome plant defenses.

Thirty-seven *P. solenopsis* CYP genes were identified, which is a relatively small number compared with the number of CYP genes in other insects. For example, genome analyses of *D. melanogaster*, An. gambiae, and *T. castaneum* identified 85, 106, and 143 CYP genes, respectively [32,33]. Sequence analysis of the 11 CYP (*PsCYP4NT1, PsCYP4G219, PsCYP6PZ1*, *PsCYP6PZ5*, *PsCYP301B1*, *PsCYP302A1*, *PsCYP305A22*, *PsCYP315A1, PsCYP353F1*, *PsCYP3634A1*, *and PsCYP3635A2*) genes showed that none of their encoded proteins were predicted to have signal peptides or transmembrane domains. The isoelectric points of the 11 CYPs were between 7.64 and 9.29, and all contained a cytochrome P450 cysteine heme-iron ligand signature sequence.

Next generation high-throughput sequencing technology generates a large amount of data, at high speed, with low cost and high efficiency, and has been widely used in research into insect molecular markers [34]. In this study, the transcriptome of *P. solenopsis* was sequenced and analyzed using Illumina sequencing technology. The antennal transcriptome of *P. solenopsis* produced a total of 47,635,589 clean reads, with an N50 value of 1297 bp. It is generally believed that the larger the N50 value, more long fragments could be obtained, while an N50 value smaller than 800 bp indicates better sequence integrity of the assembly. The base Q30 value was 92.03%, and the Q30 was above 80% [35]. These results indicated that the basic requirements of transcriptome analysis were met in terms of assembly quality and length of sequencing data, laying the foundation for further exploration of important functional genes. This study used the Nr, Nt, Pfam, SwissProt, and GO databases to perform BLASTX alignment analysis on the obtained unigenes. Through homologous sequence comparison within the Nr database, *Bemisia tabaci* came up more often than other species because it is the most closely related one to *P. solenopsis*. Sequencing analyses of most insect transcriptomes have shown that the annotated unigenes are most similar to similar and closely related species. For example, the percentage of genes annotated from the *Tenebrio molitor* antenna transcriptome in *Tribolium castaneum* was 90.81% [36]. The *Grapholita molesta* antenna transcriptome annotated the most sequences in *Danaus plexippus*, 52.2% [37]. In the GO analysis, (Figure 2), although the main categories were nucleus, cytoplasm, and integral components of membranes, we also identified annotations in iron ion binding, electron carrier activity, heme binding, and oxidation-reduction process. In the eggNOG classification, 33% of the genes were included (Figure 3). The existence of unannotated unigenes is related to the short length of the spliced fragments, the lack of genomic information, and the lack of genetic information [38,39]. In addition, the results of GO, KEGG, and eggNOG classification demonstrated that the CYP genes in the *P. solenopsis* transcriptome are involved in “metabolism of terpenoids and polyketides” and “secondary metabolites’ biosynthesis, transport and catabolism,” and these genes might have multiple functions.

Phylogenetic analysis revealed that the *P. solenopsis* CYPs could be mainly classified into the mitochondrial, CYP2, CYP3, and CYP4 families (Figure 5). Many proteins are involved in the ecdysteroids metabolic pathway in the CYP2 and mitochondrial families, including CYP302A1, CYP301A1, and CYP314A1 from the mitochondrial clan, and CYP306A1, CYP18A1, and CYP307A1 from the CYP2 clan [12]. Contrastingly, proteins in the CYP3 and CYP4 clans are more involved in the detoxification of a variety of pesticides and plant allelopathic substances, as are the CYP6 family proteins [35]. Additionally, we found five new families of P450 genes: PsCYP3633A1 and PsCYP3633A2; PsCYP3634A1; PsCYP3635A2; PsCYP3636A1; and PsCYP3638A1, PsCYP3638A2, and PsCYP3638B1. Interestingly, the PsCYP3634A1, PsCYP368A1, PsCYP3638A2, PsCYP3638B1, and PsCYP4 clans clustered together, and the PsCYP3633A1, PsCYP33A2, PsCYP3635A1, PsCYP3635A2, and CYP6 clans clustered together. We suspect that the genes of these new families may have evolved from the CYP4 and CYP6 clans respectively. This classification of CYP genes from *P. solenopsis* into different clans suggests marked functional diversity among them. This diversity may lead to better niche adaptation by the insects [40].

There are differences in the expression levels of cytochrome P450 genes in different developmental stages, tissues, and organs of insects, suggesting their different functions [41]. Studies have shown that *CYP4D1* gene expression of *Mayetiola destructor* increases with advancing age, and its expression in the sixth instar larvae is the highest. After developing into pupa, *CYP4D1* expression decreases rapidly and then increases slightly in the adult stage [42]. Similarly, the expression of the *CYP4H34* gene in *Culex quinquefasciatus* increased gradually from the egg stage to the late larva stage, before decreasing sharply in the pupal stage and remaining low in the adult stage [43]. The qPCR results of the 11 P450 genes in *P. solenopsis* at different developmental stages indicated that most of the genes reached their highest expression level in the nymph stage, and their expression level in the adult stage was relatively low (Figure 6a–c). We speculated that *P. solenopsis* needs to constantly adapt to the changing environment, including host selection pressure, during the growth and development of nymphs to adults. The high expression of the cytochrome P450 genes at this stage reflects the fact that the worm needs to upregulate the expression of cytochrome P450s to meet its vigorous physiological and metabolic needs. Therefore, expression of the 11 P450 genes might be necessary at each stage of development of *P. solenopsis*, and could be involved in the physiological metabolism of cells and organs during the growth and development of the insect on *P. solenopsis*.

To resist the feeding of polyphagous herbivorous insects, host plants have evolved a defense mechanism involving various methods, such as morphology, biochemistry, and molecular regulation. Among them, plant secondary metabolites play an important role in the plant defense against insects. Plants exert adverse and even toxic effects on the feeding, growth, and the reproduction of herbivorous insects via secondary metabolites, thereby exerting direct and indirect defense effects. For example, the allelochemicals furan coumarin, flavonoids, alkaloids, chlorogenic acid, gossypol, and hydrazine can be used as insect biotoxins and insect repellents [44,45]. The main cotton secondary metabolites, gossypol and hemialdehyde, have insecticidal activity [46]. Cytochrome P450s play an important role in the interaction between insects and their hosts. The insects’ detoxification-metabolic system can metabolize those plant secondary metabolites. Therefore, when the insects feed on host plants containing the secondary metabolites, the changes in insect detoxification metabolic enzymes and related metabolic abilities can allow the insects adapt to host plant defense. In the midgut of *Helicoverpa armigera*, gossypol could induce the overexpression of cytochrome P450 genes *CYP9A12*, *CYP321A1*, *CYP6AE11*, *CYP9A14*, and *CYP6B7*. However, only the *CYP6B6* gene was overexpressed in response to quercetin, tannic acid, and other plant secondary metabolites [47,48]. For the same insect, the cytochrome P450 genes induced by different plant sub-metabolites are also different. The results showed that majority of the 11 CYP genes were expressed at their highest levels on tomato or cotton (Figure 7a–d). We speculated that feeding on plant secondary metabolites could cause changes in insect detoxification enzymes and related detoxification mechanisms, thereby enhancing the ability of insects to metabolize plant secondary metabolites, and allowing insects to adapt to host plant defense mechanisms. Therefore, some secondary metabolites present on tomatoes and cotton might induce the *P. solenopsis* CYPs to detoxify the plant sub-metabolites, providing the insect with resistance to these substances and allowing them to better adapt to tomato and cotton. This may also be one of the reasons for the wide host range of *P. solenopsis*. Further research is needed to determine how *P. solenopsis* CYPs adapt to specific hosts.

## 5. Conclusions

In conclusion, the sequence characteristics, signal peptides, isoelectric points, and expression profiles of 11 CYP genes of *P. solenopsis* were analyzed. We found that some CYP genes showed marked differences in their expression levels after feeding on three hosts, suggesting that those genes encode proteins that might be involved in detoxification of allelochemicals produced by different host plants. To determine the functions of these genes, further research is required.

## Figures and Tables

**Table 1:** QPCR Primers for *Phenacoccus solenopsis* CYP genes.

Gene Name	Primers (5′–3′)	Product Size (bp)	GenBank Accession Number
*CYP315A1*	F: ACCGTTCATTGCTCGCTATT R: CCCATACGGCAAAGTAGCAT	211	MK862557
*CYP302A1*	F: TGGCAGCGGATATGTTATTG R: TCCTCGGTTATCGTGGATTC	151	MK862558
*CYP6PZ1*	F: TGCATAGCTGAACGATTTGC R: AGCCAAATGCCATTACGAAC	153	MK862559
*CYP301B1*	F: AGAAAAACCACATCCGTTCG R: GGCTGGACGCTATATTCGAG	162	MK862560
*CYP4G219*	F: TCGCCAGAATACAGGCTCTT R: TGCACGTCGAATTCTCTGTC	203	MK862561
*CYP4NT1*	F: CAGGACAAAAATGGCATTCA R: TGGGAATGAAGCTGGTATCC	217	MK862562
*CYP305A22*	F: GAAGCGTTGCTCCTTGAATC R: TTGCTGGTCGTAGTGAATCG	153	MK862563
*CYP3635A2*	F: CTCCCAGATGGTTTTGTCGT R: CTCCGAAAGGCAGAAAACAG	166	MK862564
*CYP3634A1*	F: ATTGTTTACTGGTCCAATGC R: TCGTTCCAATCTAATTCCAC	237	MK862565
*CYP353F1*	F: CCCGGTCAAAGTTTTGTCAT R: TCATCAACAACGGCGATAAG	199	MK862566
*CYP6PZ5*	F: CCGGAACATTTTACCGAAGA R: AGTTCGCATTTCAGCCAGAT	242	MK862567
*α-Tubulin*	F: CTGGTAAACACGTTCCCCGAG R: TGTAATGACCGCGAGCGTAG	152	KJ909508

**Table 2:** Characteristics of 37 CYP genes in *P. solenopsis.*

NO.	Clan/Gene Name	Protein Size (aa)	Gene Length (bp)	GenBank Accession Number
	CYP2 clan (3)			
1	PsCYP18A1	532	2343	MK875641
2	PsCYP303A1	500	1944	MK875642
3	**PsΥCYP305A22** *	516	1948	MK862563
	CYP3 clan (14)			
4	PsCYP6PU1	521	1834	MK875659
5	PsCYP6PV1 *	481	1608	MK875647
6	PsCYP6PW1 *	510	1613	MK875662
7	PsCYP6PX1	513	1756	MK875659
8	PsCYP6PY1	523	1699	MK875653
9	**PsCYP6PZ1**	478	1901	MK862559
10	PsCYP6PZ2 *	529	1748	MK875651
11	PsCYP6PZ3 *	536	1804	MK875658
12	PsCYP6PZ4 *	525	1643	MK875646
13	**PsCYP6PZ5**	526	3247	MK862567
14	PsCYP6PZ6 *	512	1814	MK875664
15	PsCYP6QA1	496	1718	MK875656
16	PsCYP6QB1	515	1868	MK875661
17	PsCYP6QD1 *	343	1044	MK875657
	CYP4 clan (7)			
18	**PsCYP4G219**	576	2425	MK862561
19	**PsCYP4NT1**	514	1747	MK862562
20	PsCYP4NU1	487	2550	MK875655
21	PsCYP4NV1	497	1759	MK875652
22	PsCYP4NV2*	470	1506	MK875645
23	PsCYP380C41	514	1763	MK875643
24	PsCYP380H1	531	1664	MK875665
	Mito. Clan (5)			
25	PsCYP301A1	436	1569	MK875648
26	**PsCYP301B1**	529	2167	MK862560
27	**PsCYP302A1**	490	1596	MK862558
28	**PsCYP315A1**	470	2242	MK862557
29	**PsCYP353F1**	457	1593	MK86256
	New Clan (8)			
30	PsCYP3633A1	500	1944	MK875654
31	PsCYP3633A2 *	500	1620	MK875663
32	**PsCYP3634A1**	520	2021	MK862565
33	**PsCYP3635A2**	496	1636	MK862564
34	PsCYP3636A1	504	3678	MK875644
35	PsCYP3638A1	502	1824	MK875649
36	PsCYP3638A2	514	1925	MK875650
37	PsCYP3638B1	478	1647	MK875640

Notes. * These CYP sequences are incomplete. In bold type are the 11 genes studied in this study.

**Table 3:** Sequence information for 11 *P. solenopsis* CYP genes.

Gene Name	cDNA Full Length (bp)	Length of 5′ UTR (bp)	Length of 3′ UTR (bp)	Isoelectric Point
*PsCYP4NT1*	1747	30	175	8.54
*PsCYP4G219*	2425	270	427	7.64
*PsCYP6PZ1*	1901	176	291	8.88
*PsCYP6PZ5*	3247	69	1600	8.87
*PsCYP301B1*	2167	154	416	9.21
*PsCYP302A1*	1596	36	90	9.24
*PsCYP305A22*	1948	0	400	8.74
*PsCYP315A1*	2242	381	451	8.99
*PsCYP353F1*	1593	177	45	9.29
*PsCYP3634A1*	2021	97	364	8.41
*PsCYP3635A2*	1636	51	97	8.42

UTR: Untranslated Region.

## Supplementary Materials

The following are available online at https://www.mdpi.com/2075-4450/10/9/264/s1, Figure S1: Nucleotide and deduced amino acid sequences of CYPs; Table S1: Summary of the *Phenacoccus solenopsis* transcriptom; Table S2: Annotation results from the statistics of *P. solenopsis*; Table S3: BLASTX best hit results of the CYP genes of *P. solenopsis*; Table S4: *CYP* gene family proteins used for the phylogenetic analysis deduced amino acid sequences with homologues from other species of insects deposited in the NCBI data.
