# Supplementary material for: Expression Patterns, Molecular Characterization, and Response to Host Stress of CYP Genes from Phenacoccus solenopsis (Hemiptera: Pseudococcidae)
Source: Insects. 2019 Aug 22;10(9):264. doi: 10.3390/insects10090264 (PMC6780606; doi:10.3390/insects10090264)
Supplement: Supplementary file 1 [file insects-10-00264-s001.zip › insects-560348-suppl-proof done/supplymentary materials/Figure S1.pdf]

Suppl. 4

A. Nucleotide and deduced amino acid sequences of PsCYP301B1 from *P. solenopsis*

1	ATGCTCAAATTAATTACTGCCGTTAAAAAAAAGCAAATATCTCAATGTAGAAATCGTTTC
1	M L K L I T A V K K K Q I S Q C R N R F
61	ACACTTTCGATGAACGGATCACCCCAGATCATTTGTGGGCCTTCGATACTGAGCAATGAG
21	T L S M N G S P Q I I C G P S I L S N E
121	ATACGACCATATGAAACAATACCTGGACCAAAGCCTATACCGTTGTTGGGGAACAGCTGG
41	I R P Y E T I P G P K P I P L L G N S W
181	AGATTTTTACCGTTCATAGGTCAATACAAATTAGAAGAAATAGATTTGCTTTCCAAAGCA
61	R F L P F I G Q Y K L E E I D L L S K A
241	CTTCATGAACAATTTGGTGATATCGTGAAGATAGAAGGAATATTGGGACGGCCGGATATG
81	L H E Q F G D I V K I E G I L G R P D M
301	GTGTTCCTGTACGATGCGGATGAAATTGAAAAAATTTTTCGTAATGAAGACGTTTTGCCA
101	V F L Y D A D E I E K I F R N E D V L P
361	TTGAGACCATCTATGCCATCTCTTAGTTACTACAAACATACACTTCGGAAAGGTTTTTTT
121	L R P S M P S L S Y Y K H T L R K G F F
421	GGAGATATCAGCGGAGTGATCGCAGTGCATGGACCTAAATGGTTGGAATTTCGTTCAAAA
141	G D I S G V I A V H G P K W L E F R S K
481	GTGCAACAACCAGTTCTGCAACCACGTATTGTTAAACTGTATGTACGTGATATTGAAGAG
161	V Q Q P V L Q P R I V K L Y V R D I E E
541	ATCGCCAATGCCTTCATTTCTAGAATTAAAAGGCGTCGAAGTATTAATGACGAAGTTGGT
181	I A N A F I S R I K R R R S I N D E V G
601	GCAGATTTTTTGAACGATATTCATCTTTGGTCTTTAGAAAGTATTGGAAAAATAGCGTTA
201	A D F L N D I H L W S L E S I G K I A L
661	GATGTACGATTTGGTTGCCTGGACAACGTGATTGCGAATGAAGACACGCAATTATTTATT
221	D V R F G C L D N V I A N E D T Q L F I
721	GATGCGGTGAATACATTTTTCAAGAATGTTGGCGTATTAGAATTGAAAATACCATTTTGG
241	D A V N T F F K N V G V L E L K I P F W
781	AAAGTTTTCAACACCCCTACTTGGAAGAAATATGTTCATTCCTTAGACACCATTACCAGC
261	K V F N T P T W K K Y V H S L D T I T S
841	ATCACCATGAAGTACGTAGCAGAAGCGCAAAATTCATCATACAATAGTTGCTCATCATTT
281	I T M K Y V A E A Q N S S Y N S C S S F
901	TTAACAAAGAGTAATTCTTCACTTCTTCAACGAGTCCTTGCCTTAGATCCGAGCAATCCG
301	L T K S N S S L L Q R V L A L D P S N P
961	AAATTGGCCGCAGTTCTAGCATTGGATATGTTTCTAGTTGGAATTGATACGACGTCGGCT
321	K L A A V L A L D M F L V G I D T T S A
1021	GCGGTGGCATCAGTTTTATATCAACTGAGTCAAAATCCAGACAAACAGCAAATATTATAT
341	A V A S V L Y Q L S Q N P D K Q Q I L Y
1081	GATGAAATAAATAGAGTTTTACCGAAATTGGATGACGAATTAACCATGGAAAAAATCGAC
361	D E I N R V L P K L D D E L T M E K I D
1141	AAACTTGTTTACTTAAAAGCATTTATAAAAGAAACTTTGAGATTACATCCTGTAGTGCTA
381	K L V Y L K A F I K E T L R L H P V V L
1201	GGTAATGGAAGAACATTGACTAAAGAAACAGAAATTTGTGGTTTTAAAGTTCCTAAAGGA
401	G N G R T L T K E T E I C G F K V P K G
1261	GTTCAAGTTGTATTTCAACATTTTGTTATGAGTAACTCTGAACGTTATTTCAAATGCGCC
421	V Q V V F Q H F V M S N S E R Y F K C A
1321	AACAATTTCGAGCCAGAAAGATGGTTGAAAAACAATTTAGAAAAACCACATCCGTTCGTA
441	N N F E P E R W L K N N L E K P H P F V
1381	TCATTACCTTTTGGCTTTGGAAAACGCATGTGCTTGGGAAGAAGATTCGCAGAACTTGAG
461	S L P F G F G K R M C L G R R F A E L E
1441	ATACAAGTCCTCATAACCAAGATGGTACAAAATTTCTGCGTCGACTTCAGACACAAAAAA
481	I Q V L I T K M V Q N F C V D F R H K K
1501	CTCGAATATAGCGTCCAGCCGATGTACTTGCCCAAAGGAGATTTAACCTTTACATTTAGG
501	L E Y S V Q P M Y L P K G D L T F T F R
1561	AATCGCGAGAAAAATAAAAATCAATGA
521	N R E K N K N Q *

B. Nucleotide and deduced amino acid sequences of PsCYP302A1 from *P. solenopsis*

```
1      ATGACTTTTCGATGTGGAAAAGTACGTAATTTTTCCACCGAACTGTCCCCAAAACCTTTT
1       M  T  F  R  C  G  K  V  R  N  F  S  T  E  L  S  P  K  P  F
61     AATGAGATACCAGGGCCAAAATCGTTGCCTATAATCGGAACTTTGTGGCAGTACTTGCCG
21      N  E  I  P  G  P  K  S  L  P  I  I  G  T  L  W  Q  Y  L  P
121    CTGATTGGAAAATATAAATTCGATAGACTACACTGGAATGGGTTACTGAAACTGAAAGAA
41      L  I  G  K  Y  K  F  D  R  L  H  W  N  G  L  L  K  L  K  E
181    TTCGGTCCTCTAGTCCGAGAAGAAATGATTCCCGGTCTACCAGTGGTATGGGTGTTCAAA
61      F  G  P  L  V  R  E  E  M  I  P  G  L  P  V  V  W  V  F  K
241    CCAGAAGACATCGAAACGGTATACCGAAACGAGGGGAGATACCCAGAACGTCGAAGTCAC
81      P  E  D  I  E  T  V  Y  R  N  E  G  R  Y  P  E  R  R  S  H
301    CTTGCTTTACAAAAATATCGTCTCGATAGACCGGACGTATATAATTCCGGAGGTCTTCTT
101     L  A  L  Q  K  Y  R  L  D  R  P  D  V  Y  N  S  G  G  L  L
361    CCAACCAACGGTCCGGAATGGTGGCGTTTACGGAGGATATTTCAAAAAGATTTAAATAAA
121     P  T  N  G  P  E  W  W  R  L  R  R  I  F  Q  K  D  L  N  K
421    ATTCAAAACGTTCGCTTATACTTGTCGAAATCGGACGAAATTATCAAGAATTTTTTACAA
141     I  Q  N  V  R  L  Y  L  S  K  S  D  E  I  I  K  N  F  L  Q
481    CGCAGAATTCCCAAATACGAAAACGATTTCGCGCCAGAATTATCGCGATTATATTTAGAA
161     R  R  I  P  K  Y  E  N  D  F  A  P  E  L  S  R  L  Y  L  E
541    CTGACTGGTTTAGTGGCGTTCGACGAACATTTGGGTAGTTTTAGCGATACGCAAATGGCC
181     L  T  G  L  V  A  F  D  E  H  L  G  S  F  S  D  T  Q  M  A
601    CCTAGTTCTCCAATGTCAAGGTTGATTGATTCAACGGCTTTTATAAATAGCTGCGTATTA
201     P  S  S  P  M  S  R  L  I  D  S  T  A  F  I  N  S  C  V  L
661    CGTACCGATAACGGTCCTCAGCTGTGGAGGAAATTTAACACACCGTTGTATAAAAAATTT
221     R  T  D  N  G  P  Q  L  W  R  K  F  N  T  P  L  Y  K  K  F
721    TGCGAAGCTCATTTATGCTTGGAAACAGTTGCCAAACATTTTGTATACAAGAAAATGCGC
241     C  E  A  H  L  C  L  E  T  V  A  K  H  F  V  Y  K  K  M  R
781    AAGCTTACAGAGGAACGTGGCAATCATCAATATTCTTTGTTAGAGCAATATCTACTCAAC
261     K  L  T  E  E  R  G  N  H  Q  Y  S  L  L  E  Q  Y  L  L  N
841    AAAGAGCTCGATACTAAAGATGTGATCGGAATGGCAGCGGATATGTTATTGGCTGGCATT
281     K  E  L  D  T  K  D  V  I  G  M  A  A  D  M  L  L  A  G  I
901    GATACCACCGCCTATACGAGTTGTTTCGGATTGTATCATATTTCCAAAAATGTTGACAAA
301     D  T  T  A  Y  T  S  C  F  G  L  Y  H  I  S  K  N  V  D  K
961    CAAGAAAAAATGTTCAAAGAGCTGGAATCGTTGATGAACGCAGAATCCACGATAACCGAG
321     Q  E  K  M  F  K  E  L  E  S  L  M  N  A  E  S  T  I  T  E
1021   GAAATCCTAAACCAGGCAGTCTATACGAAAGCAGTTATGAAAGAGATATTCAGGATGAAT
341     E  I  L  N  Q  A  V  Y  T  K  A  V  M  K  E  I  F  R  M  N
1081   CCGATATCTGTAGGCATTGGTAGAATTTTAGCCAAAGATGCTGTGTTATCTGGGTATCTA
361     P  I  S  V  G  I  G  R  I  L  A  K  D  A  V  L  S  G  Y  L
1141   GTGCCAGCGAAGACCATTGTTGTAACTCAAAACCAAGTGACTTGCCGATTACCAGAATAT
381     V  P  A  K  T  I  V  V  T  Q  N  Q  V  T  C  R  L  P  E  Y
1201   TTTCATAAACCGAACGAATTTATTCCGGAAAGATGGATCAAAGATCATGCACTTTATGAA
401     F  H  K  P  N  E  F  I  P  E  R  W  I  K  D  H  A  L  Y  E
1261   AACGTCCATCCTTATTTAGTTCTACCATTTGGTCACGGTCCAAGAACTTGTATAGCTAGG
421     N  V  H  P  Y  L  V  L  P  F  G  H  G  P  R  T  C  I  A  R
1321   AGATTGGCTGAACAACATTTTCAGTTGTTTTTGATGAATGTGATTAGACGATACAGAATT
441     R  L  A  E  Q  H  F  Q  L  F  L  M  N  V  I  R  R  Y  R  I
1381   ACTTGGAAAGGAGATCATTTAGATTGTAAATCATTACAGATCAATAAGCCGGATAAAAGC
461     T  W  K  G  D  H  L  D  C  K  S  L  Q  I  N  K  P  D  K  S
1441   GTCGATATTGTTTTTGAAAAACGATACTGA
481     V  D  I  V  F  E  K  R  Y  *
```

C.Nucleotide and deduced amino acid sequences of PsCYP353F1 from *P. solenopsis*

```
1       ATGAAACTACTAAGAAATCTGGGTAAAGATGTGCGACCATTTCGTGAAATACCTTCTCCA
1        M  K  L  L  R  N  L  G  K  D  V  R  P  F  R  E  I  P  S  P
61      AAAGCATATCCGATCATCGGACATGCATATTTATTTTCCGGCAAAGGTCCCTATCACAAA
21       K  A  Y  P  I  I  G  H  A  Y  L  F  S  G  K  G  P  Y  H  K
121     GATAAATTAACGGAAGCAGCTTACGATCTCAGTAAAAAATACGGTCCTGTTTTCTATCTC
41       D  K  L  T  E  A  A  Y  D  L  S  K  K  Y  G  P  V  F  Y  L
181     AAATTTCCCGGTCAAAGTTTTGTCATTACTACCGATGCGGAGAATGCTTCAAAAATATTC
61       K  F  P  G  Q  S  F  V  I  T  T  D  A  E  N  A  S  K  I  F
241     CAAAATGAAGGAAAACTTCCTAATCGCGTTACATTACCGGCTTTAGCATTATACCATGAA
81       Q  N  E  G  K  L  P  N  R  V  T  L  P  A  L  A  L  Y  H  E
301     AGATCTTTTGGTGCCGTTGGAGTAGTTCCAGACAACGGGCCAAACTGGTATAAATTGAGG
101      R  S  F  G  A  V  G  V  V  P  D  N  G  P  N  W  Y  K  L  R
361     AAAGCCTTATCGCCGTTGTTGATGAAAACCTACATTTCTAAATTTATCAATGAGAATCAA
121      K  A  L  S  P  L  L  M  K  T  Y  I  S  K  F  I  N  E  N  Q
421     CTCATCGCCGATAAATTTGTCGAATATATTAGAAAAAACAAGGATAAAAATAACACACTA
141      L  I  A  D  K  F  V  E  Y  I  R  K  N  K  D  K  N  N  T  L
481     ATCGATCTATGTTCGCATATGTATCGGTATTCCATTGAATGTATGTCAATCGTGTGCCCA
161      I  D  L  C  S  H  M  Y  R  Y  S  I  E  C  M  S  I  V  C  P
541     GGAAGACGTCTTACCAATGAAGAAGATCTACGTAAAATCATCGAAGGTAGTTGTACATTC
181      G  R  R  L  T  N  E  E  D  L  R  K  I  I  E  G  S  C  T  F
601     ATGGATGGACTCTATCGTACTTATTCGGAGCCTCCTGTGTGGAAATTTATCAAAACTAAA
201      M  D  G  L  Y  R  T  Y  S  E  P  P  V  W  K  F  I  K  T  K
661     GGATACAGAAAATTGGAACGAGGGCATAATATAATCAATAGATTCATGTACAAAGAAATG
221      G  Y  R  K  L  E  R  G  H  N  I  I  N  R  F  M  Y  K  E  M
721     GAGAAGCCAATCAATACGTGGAACGATTCCTTTATGAAAGTTCTATTTCAAGATTCTGAT
241      E  K  P  I  N  T  W  N  D  S  F  M  K  V  L  F  Q  D  S  D
781     TTAACTCGAAACCAAGCTCGTGTTTTATCCACTGAAGTATTTTTATCAGGAATTGATTCG
261      L  T  R  N  Q  A  R  V  L  S  T  E  V  F  L  S  G  I  D  S
841     ATATCCACCGCTATCACCATGACTTTGTATTATCTTTCCAAAAATGAAAAATGTCAACAT
281      I  S  T  A  I  T  M  T  L  Y  Y  L  S  K  N  E  K  C  Q  H
901     GAAGCTCTAAATGATGTGAAAAATGGCTCTCTGTCATACTTAAGAGCCTGTTTCAAAGAA
301      E  A  L  N  D  V  K  N  G  S  L  S  Y  L  R  A  C  F  K  E
961     ACATTAAGATTATCAGCAACTGCTGGCGGTACCACTAGAGCACTTCCTGCTGATGTAGTT
321      T  L  R  L  S  A  T  A  G  G  T  T  R  A  L  P  A  D  V  V
1021    ATGAATGATTACAACATTCCTGCAAATACTTACATTACCGCTTTCCATTCTGCAATGTCA
341      M  N  D  Y  N  I  P  A  N  T  Y  I  T  A  F  H  S  A  M  S
1081    CTTATGGATGAGTATTTTGAAGATGCTGATCTTTATAAACCAGAAAGGTGGTTGCATTCG
361      L  M  D  E  Y  F  E  D  A  D  L  Y  K  P  E  R  W  L  H  S
1141    GAGAAGGTGCAAAAGAAAAACGAATTCAGTAATTTTGCCTCTTTACCTTTCGGACATGGA
381      E  K  V  Q  K  K  N  E  F  S  N  F  A  S  L  P [F  G  H  G]
1201    CCGAGAATGTGCCCTGGGAGATGGTTATCTGAACAAGAAAGTGTAATCTTGTTGAGCGCT
401     [P  R  M  C  P  G] R  W  L  S  E  Q  E  S  V  I  L  L  S  A
1261    ATATTAAAATCGTATAAATTAGAATCAGCGACGCCATCTCGAGTCGGTATGATATATAGA
421      I  L  K  S  Y  K  L  E  S  A  T  P  S  R  V  G  M  I  Y  R
1321    ATGAATCGTATTCCAGAGCGTTCGATCGATATAAAGTTTATAGATAGATAA
441      M  N  R  I  P  E  R  S  I  D  I  K  F  I  D  R  *
```

D. Nucleotide and deduced amino acid sequences of PsCYP3634A1 from *P. solenopsis*

```
1       ATGTCAAAAATACGTGATGAAATCATAACTTATTCGACATCGGTGAAAATTGGCCTACTA
1        M  S  K  I  R  D  E  I  I  T  Y  S  T  S  V  K  I  G  L  L
61      ACTATTCTAATTGTGTTATTATGGAAATATTTAATGAAATTATTCCGTCGTGATCATCGT
21       T  I  L  I  V  L  L  W  K  Y  L  M  K  L  F  R  R  D  H  R
121     TTGGTGACATTTGCTGAAACTCTACCCGGACCCCCAACGATACCTATAATAGGAAACGGT
41       L  V  T  F  A  E  T  L  P  G  P  P  T  I  P  I  I  G  N  G
181     TTAGATTTCTACTATGGAGGCGTCCATGCAATGAAGAAGGTAATGGGATATACCGAAAAA
61       L  D  F  Y  Y  G  G  V  H  A  M  K  K  V  M  G  Y  T  E  K
241     TATGGCCATATATATAGGCTATGGTTCGGTAAATTTTTAGTCGTTGGATTATCGAAAGTT
81       Y  G  H  I  Y  R  L  W  F  G  K  F  L  V  V  G  L  S  K  V
301     GAAGATTTAGAGGATGTATTTATGAATTCAAAAACGATGGGAAAACCGGAAATATTTCGA
101      E  D  L  E  D  V  F  M  N  S  K  T  M  G  K  P  E  I  F  R
361     CCATTTCAGGATTATTGGGGTGATGGATTGTTTACTGGTCCAATGCATATTTGGAAAAAA
121      P  F  Q  D  Y  W  G  D  G  L  F  T  G  P  M  H  I  W  K  K
421     AATAGAAAACGGTTTCAGCCCATTTTTGGAAATAATATAATGGGCTTATACACTCCAGAA
141      N  R  K  R  F  Q  P  I  F  G  N  N  I  M  G  L  Y  T  P  E
481     TTCAATGATAAAATTTATAAAGTCGTTGATATGATGAAAACGTTGTTGAATGGACCAGAA
161      F  N  D  K  I  Y  K  V  V  D  M  M  K  T  L  L  N  G  P  E
541     TTTGATGCTTGGGATTATTTACCTTATTTATCATTTGATATCATTACTAAAACCTCATTA
181      F  D  A  W  D  Y  L  P  Y  L  S  F  D  I  I  T  K  T  S  L
601     GATGTGGAATTAGATTGGAACGATAAATCTGTGTTACAGTTTCACAATTCTGTAAAAACG
201      D  V  E  L  D  W  N  D  K  S  V  L  Q  F  H  N  S  V  K  T
661     GGAATGGAAACTAGTTTATTAAAAATGCTTCGGCCATGGTTACATATAAAATTCTTGAGA
221      G  M  E  T  S  L  L  K  M  L  R  P  W  L  H  I  K  F  L  R
721     GATTTATTATACAAAAAACAAGTTGAAAAAGTCCAGGGTAGTATGTTAAAATTTTCGCAA
241      D  L  L  Y  K  K  Q  V  E  K  V  Q  G  S  M  L  K  F  S  Q
781     CAGACTATCCAAAATAAGTTGAATATAATTAAAGATGATATATTACGACGCAAAAATATA
261      Q  T  I  Q  N  K  L  N  I  I  K  D  D  I  L  R  R  K  N  I
841     ATTAATCAAGATCCAAATGAAGTGATATCATTAAAATCATTTTTAACCACTGCTTATGAG
281      I  N  Q  D  P  N  E  V  I  S  L  K  S  F  L  T  T  A  Y  E
901     ATACAAGAGGAAGTAAATAATGAAAAACTTATGCACGATGAAATCATTACCATGATAACC
301      I  Q  E  E  V  N  N  E  K  L  M  H  D  E  I  I  T  M  I  T
961     GGGGGTACTGATACCACAGCGATAACAAATTCGTTTTTCCTACTGGCAGTTGCTATTTAT
321      G  G  T  D  T  T  A  I  T  N  S  F  F  L  L  A  V  A  I  Y
1021    CAAGATATACAGAGTAAATTATACGATGAAATGTATGAAGTTTTCGGCGATAGTGATCGA
341      Q  D  I  Q  S  K  L  Y  D  E  M  Y  E  V  F  G  D  S  D  R
1081    TATGCTGACCACGACGATGTGAAACGATTACCATATTTAGACCAAGTGTTGAAGGAAACC
361      Y  A  D  H  D  D  V  K  R  L  P  Y  L  D  Q  V  L  K  E  T
1141    TTACGCAGATTTACTTTGGCTCCAGTCATTTTCAGAGATGTAGAAGAAGATTGCAAAATA
381      L  R  R  F  T  L  A  P  V  I  F  R  D  V  E  E  D  C  K  I
1201    GGTGGTAGACTATTTCCGGCTGGTACCGCGTTAATGATTTCTATCATAGGCGTTCATTTT
401      G  G  R  L  F  P  A  G  T  A  L  M  I  S  I  I  G  V  H  F
1261    GATCCTGAATATTATCCAAATCCGTGGAAATTCGATCCAGAAAATTTTAGTCCTGAGGCT
421      D  P  E  Y  Y  P  N  P  W  K  F  D  P  E  N  F  S  P  E  A
1321    GTGGAGAAACGTCATAAATTGACATTTCTACCATTTAGCAGTGGTCCTCGCAATTGCATT
441      V  E  K  R  H  K  L  T  F  L  P  F  S  S  G  P  R  N  C  I
1381    GGTCAAAATTACGCCATGTTGGAAATGAAATTGACATTAATCGCTTTGCTGAGACATTTC
461      G  Q  N  Y  A  M  L  E  M  K  L  T  L  I  A  L  L  R  H  F
1441    AGTTTTCATACAACTATGACAATGGACGATATAAAGATGAATATGGGATTTTTAATGACC
481      S  F  H  T  T  M  T  M  D  D  I  K  M  N  M  G  F  L  M  T
1501    AGCGTTGACGGGTATAAAATGAGCATTAAACCACGGAACAGAAAACCATCATATTTGTGA
501      S  V  D  G  Y  K  M  S  I  K  P  R  N  R  K  P  S  Y  L  *
```

E. Nucleotide and deduced amino acid sequences of PsCYP6PZ1 from *P. solenopsis*

```
1     ATGTCTCAAATCTTCCTGCTACGAACATCATTGGCCGAAATATACAGGGATATCTACAAT
1      M  S  Q  I  F  L  L  R  T  S  L  A  E  I  Y  R  D  I  Y  N
61    AAGCTGGAACCTCATAAATTCGGAGGAATATTCGCAGTCAAGAAAAAGACCATAATAATA
21     K  L  E  P  H  K  F  G  G  I  F  A  V  K  K  K  T  I  I  I
121   AGAGATCCGGAATTAATTAAAAATGTGCTCATTAAAGACTTCGAGTACTTTCATGATCGC
41     R  D  P  E  L  I  K  N  V  L  I  K  D  F  E  Y  F  H  D  R
181   GGCACCAAAGTCGATCAGGAAGTGGATCCGTTGGGATATCATTTATTCAACATGAGAGGC
61     G  T  K  V  D  Q  E  V  D  P  L  G  Y  H  L  F  N  M  R  G
241   GAAGAATGGAAAAATCTAAGAATCAAACTTACCAGCACTTTTACAACTGGGAAAATGAAG
81     E  E  W  K  N  L  R  I  K  L  T  S  T  F  T  T  G  K  M  K
301   ATGATGTTTCCCTTGGTAAACGAATGCGGCAAAAAATTAAACACCGTGCTTGCTAACTTA
101    M  M  F  P  L  V  N  E  C  G  K  K  L  N  T  V  L  A  N  L
361   CCTGACAGCGAGGGATTCGACATTAAAGATTTGGCAGCGAGATTCACAACCGACACAATT
121    P  D  S  E  G  F  D  I  K  D  L  A  A  R  F  T  T  D  T  I
421   GGCACTTGCGCTTTTGGCTTAGAAACCAATTCTTTGGATAATCCTGATTCAGAATTCAGA
141    G  T  C  A  F  G  L  E  T  N  S  L  D  N  P  D  S  E  F  R
481   CGAATGGGCAAAGCAATATTCAAGTTCAGATATCAGTCTTTAATACGAAGTGTGTGGACA
161    R  M  G  K  A  I  F  K  F  R  Y  Q  S  L  I  R  S  V  W  T
541   AGCATTCCACCAACACTAATCAAGAGATTGAAATTGGATTTTATTGAAAAAAAGATTCAA
181    S  I  P  P  T  L  I  K  R  L  K  L  D  F  I  E  K  K  I  Q
601   GATTATTTCATGGAAATTGTTGATGAAACTGTCCAGTATCGCGAAAAAAATAAAATATCC
201    D  Y  F  M  E  I  V  D  E  T  V  Q  Y  R  E  K  N  K  I  S
661   AGAAACGATTTTCTCGATTTACTCATCGCTTTAAAAAATAACACCATAATCGAAAAATTT
221    R  N  D  F  L  D  L  L  I  A  L  K  N  N  T  I  I  E  K  F
721   CAAGATACTGCTGAAACAGACGACCTAGAAAAATTTCTAGCTCAAGTAGGCGATAAACGC
241    Q  D  T  A  E  T  D  D  L  E  K  F  L  A  Q  V  G  D  K  R
781   ATCAAAAGTAACATAGATATGTCTAATGCGATGCTAGCAGCTCAATGTTTTGTTTTCTTC
261    I  K  S  N  I  D  M  S  N  A  M  L  A  A  Q  C  F  V  F  F
841   ATTGCTGGATTTGAGACCTCCTCCACCACGTTAGGATTTTTATTACTAGAACTAGCGCAA
281    I  A  G  F  E  T  S  S  T  T  L  G  F  L  L  L  E  L  A  Q
901   AATGAAGAAATTCAAAACAAGGTACGCGATGAAATTCGAGCAGTTTTGGAAAACAATGAT
301    N  E  E  I  Q  N  K  V  R  D  E  I  R  A  V  L  E  N  N  D
961   AATGAACTCACATACGATAGTATGAAACAAATGACTTACACGGATATGGCAATTGCAGAA
321    N  E  L  T  Y  D  S  M  K  Q  M  T  Y  T  D  M  A  I  A  E
1021  GCGCTCAGGAAATACCCCCCAGCAGGAATATTAATGAGAGAAGCGAATAAAAATTACAAA
341    A  L  R  K  Y  P  P  A  G  I  L  M  R  E  A  N  K  N  Y  K
1081  ATACCAAATACCAAAATAATAATTCCTGCCAATACCGCCATTATAATTCCAGTCTTTGGA
361    I  P  N  T  K  I  I  I  P  A  N  T  A  I  I  I  P  V  F  G
1141  ATACACAGGGATGCGAAGTATTACGATAACCCTGAAGAATTTCGTCCAGAAAGATTCACC
381    I  H  R  D  A  K  Y  Y  D  N  P  E  E  F  R  P  E  R  F  T
1201  GAGCGAGAAAAAGCCAAACGACCGCACTATACTTATCTACCATTTGGAGAAGGACCCAGA
401    E  R  E  K  A  K  R  P  H  Y  T  Y  L  P  F  G  E  G  P  R
1261  GTTTGCATAGCTGAACGATTTGCCAAAATGCAAGTCAAAATTGGATTAGTATACATGTTG
421    V  C  I  A  E  R  F  A  K  M  Q  V  K  I  G  L  V  Y  M  L
1321  AAAGACTTTTCTTATCATCTTTCGCCCAAAATGAAATTCCCATTGGAGTTTGAAAAAAAT
441    K  D  F  S  Y  H  L  S  P  K  M  K  F  P  L  E  F  E  K  N
1381  TTCGGACTTTTGACCGTTCGTAATGGCATTTGGCTGCAAAGAACAAAGTTATAG
461    F  G  L  L  T  V  R  N  G  I  W  L  Q  R  T  K  L  *
```

F. Nucleotide and deduced amino acid sequences of PsCYP6PZ5 from *P. solenopsis*

1 ATGTACATTTTTAGTGTATTTTTCTTATTCATTGTAACGGTGTTCCTGTACATCTATCAT
1 M Y I F S V F F L F I V T V F L Y I Y H
61 GTATTCAGAAAATCTTACAAGTTCTTCGAAGATGCTGGAATACCGTACATCAAACCGCAA
21 V F R K S Y K F F E D A G I P Y I K P Q
121 TGGATATTTGGCAATATGAAGGATGTACTATTATTCAGAAAATCCTTACTCGAAAGATAC
41 W I F G N M K D V L L F R K S L L E R Y
181 CAAGAGTTATACCATCATTTAGATCCTCATCCATACGCAGGAGTATTTCTAGCCCATAAG
61 Q E L Y H H L D P H P Y A G V F L A H K
241 CCCTCTGTTATGATACGAGATCCCGACCTAATCAAAGCTGTTCTAGTTAAAGATTTCGCA
81 P S V M I R D P D L I K A V L V K D F A
301 TATTTTCATGATCGTATGATTGCTGGTATTTGCAAGAAAACTGAACCAATAGCTAACCAA
101 Y F H D R M I A G I C K K T E P I A N Q
361 ATATCCATGATGAAAGGAGACGAATGGCGGAACCTTCGTATCAAATTGACCAGTACATTC
121 I S M M K G D E W R N L R I K L T S T F
421 AGCAGCGGAAAAATGAAATTCATGTTTCCCACATTACTCAAGTGCTCCGAAGGCATTAAA
141 S S G K M K F M F P T L L K C S E G I K
481 ACGGCGCTTGAAAAGGTATGCTCAGATTCTGAAGGATTCGAAGTTAAAGATTTATGTTCC
161 T A L E K V C S D S E G F E V K D L C S
541 AGGTATACGACTGATGTAATAGGTAATTGCGCTTTTGGTATGGAAACACATTCCCTGGAA
181 R Y T T D V I G N C A F G M E T H S L E
601 AATCCTGATTCCGAATTCAGAAAAATGGGAAAACGTGTATTATCGTTTAGATGGCAAACA
201 N P D S E F R K M G K R V L S F R W Q T
661 ATGCTACGCATGTTCTTTCCCAACATACCCAACAATTTCATTAAAATCTTCGGATTACGA
221 M L R M F F P N I P N N F I K I F G L R
721 TTTTTTGAGAGAGAAGTCAGCGAATATTTCACAAATATCGTCAAAGATGCAGTGAAGCAC
241 F F E R E V S E Y F T N I V K D A V K H
781 AGAGAACAGAATAACATAACCAGAGGTGATTTTCTAGATCTACTTATCGCTTTGAAAAAT
261 R E Q N N I T R G D F L D L L I A L K N
841 AACACCGAATTGGAGAAATTAAAAAATCAAAACGACGACAAAGACTTGATAAAATTCATG
281 N T E L E K L K N Q N D D K D L I K F M
901 TCTCAAATTGGCGATAAGGTCATAAAAAGCAAAATAGATATGACTATTGAAACAATGACT
301 S Q I G D K V I K S K I D M T I E T M T
961 GCCCAATCATTTCTCTTTTTCTTGGCTGGATTTGATCCAACCGCAACAACGCTTAGTTTC
321 A Q S F L F F L A G F D P T A T T L S F
1021 CTTCTCTTCGAATTATCACAAAATCAACAAATTCAAGAAAAACTTCGACAAGAGATCATA
341 L L F E L S Q N Q Q I Q E K L R Q E I I
1081 TCTACATTGGAAACTAATGATGGAATATTAACTTACGCAATGCTCAAAAAAATGCCTTAC
361 S T L E T N D G I L T Y A M L K K M P Y
1141 TTGGATATGGTTGTTGCGGAGACGTTAAGAAAATGGCCACTTGGGCTTATCATGCGTATA
381 L D M V V A E T L R K W P L G L I M R I
1201 TGCAATCAAAATTACAAGATTCCTGATTCAAATGTAATCATCAAAGAAGGAACCGAAGTT
401 C N Q N Y K I P D S N V I I K E G T E V
1261 ATCATTTCCGCGCAGGGTCTTCATTCTGATAAGAAATATTTTGAAAAACCAGATGAATTT
421 I I S A Q G L H S D K K Y F E K P D E F
1321 TATCCGGAACATTTTACCGAAGAAGCAAAGGCAGCTAGACCTCATTACGCCTATTTACCT
441 Y P E H F T E E A K A A R P H Y A Y L P
1381 TTCGGAGAGGGTCCTCGAAATTGTATAGCTGAACGATTTGCTAAAATGATGGTTAAAGTT
461 F G E G P R N C I A E R F A K M M V K V
1441 GGAACAATCTACTTCCTCAAAGATTTTTCTTTTGTATTATCACCAAAAACCAAATTACCA
481 G T I Y F L K D F S F V L S P K T K L P
1501 TTGGATATATTACCTTCATTTGGTAATATTGCAGTAAAAAATGGAATCTGGCTGAAATGC
501 L D I L P S F G N I A V K N G I W L K C
1561 GAACTTATACATAGATAG
521 E L I H R *

G. Nucleotide and deduced amino acid sequences of PsCYP3635A2 from *P. solenopsis*

1	ATGTATTTTTCTGTATGCCTGTGTTTGGTAATATGCATCTTGTACTTATGGCTCCGATAT
1	M Y F S V C L C L V I C I L Y L W L R Y
61	AAACTAAGCTACTGGGAGAGACGTGGAGCTCCTTCTTTACCCGTCAATTTCTTCAGCAGC
21	K L S Y W E R R G A P S L P V N F F S S
121	GAATTAAAATGGTTGGTAATGAAACAATCGCGATTCCAATACTTCAAGGATATCTACGAC
41	E L K W L V M K Q S R F Q Y F K D I Y D
181	CAATTGGCACCACATAAATATGGCGGATACAGCGTAATGCCACTTAAAATGATGGTACGA
61	Q L A P H K Y G G Y S V M P L K M M V R
241	GATCCTGAAATCATTAAATTCGTGCTGACCAAGGAATTCGAGAGTTTTTCCAGTCGCGAG
81	D P E I I K F V L T K E F E S F S S R E
301	AAACTATTCTTCACTAAGAGCGATCCGTTGACTCATTTTATTTTTAATTTAGAAGGCAAC
101	K L F F T K S D P L T H F I F N L E G N
361	GAATGGAAAGATACCAGAGCTCGATTGACTCCTGGATTTAGTTCGGGTAAATTGAAATAT
121	E W K D T R A R L T P G F S S G K L K Y
421	ATGTTTGATTTGATCGCCGAATGTGCAGACGAGATGGATAAAAGTTTGCATATCAACGAG
141	M F D L I A E C A D E M D K S L H I N E
481	GATAGGCAACTCGTCGACGTATGCAAGGTACTGAGATTGTACGTTATGAACGTAATTTTC
161	D R Q L V D V C K V L R L Y V M N V I F
541	ACCTGCACGTACGGATTGAAAGTGAATTCCATGAAAAATGGTTGCGATATATTTTTAAAA
181	T C T Y G L K V N S M K N G C D I F L K
601	ATGGCTGGTTTAATTCGTGGTGATAATGAAATTAATAGAAAATTGGCGAATTTTTCTTTT
201	M A G L I R G D N E I N R K L A N F S F
661	ATATTTCCATCCGCCATAAGACACTATATTAGGCCTTCTACTTTTGGCGAGTACTCGAGA
221	I F P S A I R H Y I R P S T F G E Y S R
721	GATTTCTTCAAGGGCATTATCATGGATTCCATCAAATACAGAGAGGAAAATGATATAAAG
241	D F F K G I I M D S I K Y R E E N D I K
781	AGGAATGATTTCTTGCAGATTTTCATGACCTTGATGAAGGCTGAAGATGAATTCAAGTGG
261	R N D F L Q I F M T L M K A E D E F K W
841	ACCATAGACAAAACATTAGCTCACGGTATTCTCTACATCATGGCTGGTTTCTCAACCACC
281	T I D K T L A H G I L Y I M A G F S T T
901	ACCAGCGCTGTCAGTTTCCTCCTATATGAAATGGCCAAACACCCAGATATGCAAAAAAAA
301	T S A V S F L L Y E M A K H P D M Q K K
961	GTCCACAAAGAACTGGATGAAATAATGGAAGAGTTCGATGGCACCTTAAATTACGAATGC
321	V H K E L D E I M E E F D G T L N Y E C
1021	GTTTCTAATACGAACTACTTGAATAAAGTTATATCAGAAACCATGAGAAAATACAGCGTT
341	V S N T N Y L N K V I S E T M R K Y S V
1081	ATATCTTCATTTTCCCGACGCTGCACCAGCACATGTACGCTCCCAGATGGTTTTGTCGTC
361	I S S F S R R C T S T C T L P D G F V V
1141	GAGAAAGGCACCGCCGTTGTGGTTTCAGTTAGCGGACTTCATCACGATCCAGCTTATTAC
381	E K G T A V V V S V S G L H H D P A Y Y
1201	CCAGATCCGTTGAAATTCGATCCAGAACGTTTTAATGAGGAAAATAAGAACGCTAGACAT
401	P D P L K F D P E R F N E E N K N A R H
1261	CCGTACTGTTTTCTGCCTTTCGGAGCTGGTCCGAGATATTGCATAGGCGAACGATTTTCG
421	P Y C F L P F G A G P R Y C I G E R F S
1321	ATGCTGATGATAAAAGCGGCAACATTCACAATTCTGAAGAAATTTTCCGTTTCTGTGTTA
441	M L M I K A A T F T I L K K F S V S V L
1381	CCGGAACATGAAATAGAGTTTGATTTTATGCCTAATAAGCTGACCACTGAAGTACCAAGT
461	P E H E I E F D F M P N K L T T E V P S
1441	ATCAAGTTACAATTCACTCATAGGCAACGTACTACAGATATTCAATGA
481	I K L Q F T H R Q R T T D I Q *

H. Nucleotide and deduced amino acid sequences of PsCYP15A1 from *P. solenopsis*

1	ATGATGTTGAAGTTGAATAGAATAAAATATGTATTTCATTACAACTACTCCACTAATGCT
1	M M L K L N R I K Y V F H Y N Y S T N A
61	AAGCCGTTTGAAGAAATACCTTCGCCAAAAGGTTTACCATTTGTTGGTACGTTATTTTCT
21	K P F E E I P S P K G L P F V G T L F S
121	TTTATCGCTGCCGGAGGAAGTACACAACTCCATGAATATATTGATAAAAGGCATCAACAA
41	F I A A G G S T Q L H E Y I D K R H Q Q
181	CTTGGAATAATTTTTCAAGAGAAATTAGGACCTATTTCGAGCGTGTTTTTAAATGATGCT
61	L G I I F Q E K L G P I S S V F L N D A
241	CATGAAATACGAAAAGTATTCGTTGGTGAAGGTAAATATCCGAAACACGTTCTTCCTGAC
81	H E I R K V F V G E G K Y P K H V L P D
301	TGCTGGCTTTTGTACAATAAAATAAAGGGATATCAGAGAGGTTTATATTTCATGGATGGA
101	C W L L Y N K I K G Y Q R G L Y F M D G
361	GAAGAATGGTTTATGTACCGCAGGTTGTTAAATGATAAACTTTTAAAACATGATTACTAC
121	E E W F M Y R R L L N D K L L K H D Y Y
421	GGACATGGAATACAGAACGATTATCTTCTACAAAAGACATTTGAAATATGGCTTCAAAAT
141	G H G I Q N D Y L L Q K T F E I W L Q N
481	TCTAAATACGGTGAAATAATTGATTTAGAAAATCAGTTATACTACCTCTCTATTATATTT
161	S K Y G E I I D L E N Q L Y Y L S I I F
541	ATGATGTCTTTTACTCTTGGACGTTCTTTTCACGATAATGTCGATAAATTTTTACCCAAT
181	M M S F T L G R S F H D N V D K F L P N
601	GTTGAAAAGTTAGCATTGGTGGTGAAAGACATCTTTAAATGGAGTGTAAAACTATCAATT
201	V E K L A L V V K D I F K W S V K L S I
661	CTGCCAGCGACTTTGGCCAGTAGTTTAAATTTACCTGTTTGGAATAATTTTGTCACTGCA
221	L P A T L A S S L N L P V W N N F V T A
721	GTTGACGAATCTATTAAACTTACCACAGATCTATTAGAAGGAGCTGAACTAAAGGCTAGT
241	V D E S I K L T T D L L E G A E L K A S
781	GATGGAATTCTCTATTTTTTATTAGACCAAAACATTCCAATAGATGTAGCGCAACGAATA
261	D G I L Y F L L D Q N I P I D V A Q R I
841	ATTGTGGATTTTTTGTTGGCTGCTGGAGATACGACTGCTTTGGCTACCCTTTGGATATTG
281	I V D F L L A A G D T T A L A T L W I L
901	TATTTGTTAGGAAGGCATAAAAATATTCAAGACGAGTTATATAGTGATATTCAAAAAAAT
301	Y L L G R H K N I Q D E L Y S D I Q K N
961	CGTGATAGTCATGAAACTCGATTATTGCGGAACATAATTAAAGAATCCATGAGATTGTAT
321	R D S H E T R L L R N I I K E S M R L Y
1021	CCGATCGCACCGTTCATTGCTCGCTATTTGCCACAGGAAACAGAAATAGCTTCCTACCTT
341	P I A P F I A R Y L P Q E T E I A S Y L
1081	ATTCCAGCTAACCATTTAATCAGCATATCGGTCTTCAACTGTAGCAGAAATGAAAAATAT
361	I P A N H L I S I S V F N C S R N E K Y
1141	TTCCCTAAAGCGAATAAATTTGAACCAAAAAGGTGGGATCGTTTGGCAAATGGAAAATAC
381	F P K A N K F E P K R W D R L A N G K Y
1201	GCTGAAGTCAATGATCCATATGCTACTTTGCCGTATGGGATGGGAGCACGATCTTGTATT
401	A E V N D P Y A T L P Y G M G A R S C I
1261	GGCAGGAATTTGTCTAAAGTACAAATCAGTATCATCATAAACAAATTAATTGCTTCGTAT
421	G R N L S K V Q I S I I I N K L I A S Y
1321	GAACTTGAAGTTCGAAATGAGGTTGGAGTGAAATTAAACATGGTTACAACTCCTTCACAA
441	E L E V R N E V G V K L N M V T T P S Q
1381	CCCATCAAACTATTCTTGAAAAAAAGATGA
461	P I K L F L K K R *

I. Nucleotide and deduced amino acid sequences of PsCYP4NT1 from P. solenopsis

```
1      ATGCAATTTACTTTGTACGAATATTCGATTTCTTATTCCGCCGCTATGTCGGTGTTTTTG
1       M  Q  F  T  L  Y  E  Y  S  I  S  Y  S  A  A  M  S  V  F  L
61     ATTTTTATTTTAACGCTTCTCGTTTCATTGCTTTGTAAACGACGTATGCAATTTTTACGA
21      I  F  I  L  T  L  L  V  S  L  L  C  K  R  R  M  Q  F  L  R
121    TATTTTGAAACAATACCAGGTCCAAGTGCTATACCGATTTTAGGAAATGCTTTACAACTA
41      Y  F  E  T  I  P  G  P  S  A  I  P  I  L  G  N  A  L  Q  L
181    ACTGGATCTCAAGCAGAATTCTTCAAGCTTGTACGACAATATGCTGAAAAATACAAAAAT
61      T  G  S  Q  A  E  F  F  K  L  V  R  Q  Y  A  E  K  Y  K  N
241    ATGTTTGTATTATGGATCGGACAAAGACCTTTCGTGTTTTTGAACAAGGCGGAAGTTATT
81      M  F  V  L  W  I  G  Q  R  P  F  V  F  L  N  K  A  E  V  I
301    CAGCCCTTACTGAATTCTAGCGTGCATATTGAAAAAAGTTTGGAATACAAACTTCTAGAT
101     Q  P  L  L  N  S  S  V  H  I  E  K  S  L  E  Y  K  L  L  D
361    CCTTTCCTTGGCACCGGATTAGTGACTAGTGCAGGACAAAAATGGCATTCACAAAGAAAA
121     P  F  L  G  T  G  L  V  T  S  A  G  Q  K  W  H  S  Q  R  K
421    CTTTTGACGCCAGTATTTCACTACAATATGCTTGAAAATTATCTCCACACTGTACTAAAA
141     L  L  T  P  V  F  H  Y  N  M  L  E  N  Y  L  H  T  V  L  K
481    GAAAGCGAAGTTTTAGTCCAGCAACTTCAAAAAGAAGTGGATCACCCATTTAACATCGTG
161     E  S  E  V  L  V  Q  Q  L  Q  K  E  V  D  H  P  F  N  I  V
541    CCCTACATGAAATTAGCAGCTATGGACATAATCTGCGAGCTAACACTAGGATACCAGCTT
181     P  Y  M  K  L  A  A  M  D  I  I  C  E  L  T  L  G  Y  Q  L
601    CATTCCCAAATCAACGCTAATCTGGAATACGTTCATGCTATTGAAGATTTAATGGCTATT
201     H  S  Q  I  N  A  N  L  E  Y  V  H  A  I  E  D  L  M  A  I
661    GCACAAAGGCGATTCATCACTCCATGGCTAAAACCAAATTTCTTGTTCAACTGCACACCA
221     A  Q  R  R  F  I  T  P  W  L  K  P  N  F  L  F  N  C  T  P
721    TATGCTGCTAGGCAGAAAAAATGTTTGGACATAGTTAATACGTTTTCAACCAAAATTATC
241     Y  A  A  R  Q  K  K  C  L  D  I  V  N  T  F  S  T  K  I  I
781    GAAAAGAAAAAAGAAGAGTACCAAAATTCGCAAAAAAACAAAGTTGCGTTGGATGTCAAT
261     E  K  K  K  E  E  Y  Q  N  S  Q  K  N  K  V  A  L  D  V  N
841    TCCAATATTTTACAAAAAAAAAAAGGAAAAGTGTTTTTAGACTTATTGTTAGAACTTTCG
281     S  N  I  L  Q  K  K  K  G  K  V  F  L  D  L  L  L  E  L  S
901    GAAAACGGATCGATTCTCACCGATGATGATATAAGAGAAGAAGTGAACACATTCATATTT
301     E  N  G  S  I  L  T  D  D  D  I  R  E  E  V  N  T  F  I  F
961    GCCGGTCACGATACTACATCGATAACTGTTTCTTGGAGTCTTTACCTGTTGGGATTTCAT
321     A  G  H  D  T  T  S  I  T  V  S  W  S  L  Y  L  L  G  F  H
1021   CCAGAAGCTCAAGAGAAAATTTTAAAAGAATTAAATGAAAAAATACCGGATTTCGGAAGT
341     P  E  A  Q  E  K  I  L  K  E  L  N  E  K  I  P  D  F  G  S
1081   TCTTCATTAAAAACATCAGATATTGGTAGTCTGGAATATTTGGAATGCTGTATCAAAGAA
361     S  S  L  K  T  S  D  I  G  S  L  E  Y  L  E  C  C  I  K  E
1141   GTCTTAAGACTATATCCTCCAGTTCCTTTAATAGCAAGACATATAACTTCACCATTAGAA
381     V  L  R  L  Y  P  P  V  P  L  I  A  R  H  I  T  S  P  L  E
1201   ACGATGAATCAGATAATACCACCAGGAACTAGCGCATTAATCAATGTATACTCCTTACAT
401     T  M  N  Q  I  I  P  P  G  T  S  A  L  I  N  V  Y  S  L  H
1261   AGAGATCCACAAAACTTTCCTAATCCTTCTGAGTTCATTCCCGAAAGGTTTTCAGCAGGA
421     R  D  P  Q  N  F  P  N  P  S  E  F  I  P  E  R  F  S  A  G
1321   CAGTCAGTTGAAAAGAATCCATTTTGTTATATTCCATTTAGCGCTGGCCCGCGAAACTGT
441     Q  S  V  E  K  N  P  F  C  Y  I  P  F  S  A  G  P  R  N  C
1381   ATCGGGCAAAAATTAGCCATTCAAAGCGTCAAGATAATTTTAGCAAATATTATCAAGAAT
461     I  G  Q  K  L  A  I  Q  S  V  K  I  I  L  A  N  I  I  K  N
1441   TACAAAATTAAGACAATGCAGAAAGAAAAAGATTTGAGATTAATTTCCGAAGTAGTTTTA
481     Y  K  I  K  T  M  Q  K  E  K  D  L  R  L  I  S  E  V  V  L
1501   ACCAACGAAGGTGGAATTAACATTACCATCGAGAAAAGATGA
501     T  N  E  G  G  I  N  I  T  I  E  K  R  *
```

J. Nucleotide and deduced amino acid sequences of PsCYP4G219 from *P. solenopsis*

1	ATGGCAGTATCATTAGAGACAGTCAATGTTTTAAGATTATTCTCATCCACAACAACCTTC
1	M A V S L E T V N V L R L F S S T T T F
61	TTCTACCTCTTAATACCGGCGCTGACGCTATGGTACATTTATTTTAGAATCAGTAAAAAG
21	F Y L L I P A L T L W Y I Y F R I S K K
121	AGATTTTACGAATTACTATCGAAAATCGACGGTCCGTCCGGATTACCGCTTTTGGGAAAC
41	R F Y E L L S K I D G P S G L P L L G N
181	GCTTTAGAATTCATCGGCGATCCGCATACTACTTTTAAAATTCTTTACGAGAGGAGTTTC
61	A L E F I G D P H T T F K I L Y E R S F
241	GAATTCGAATTCGGAAAACCGATTAAGATTTGGATTGGACCAAGAGTGGTGGTGTTCTTG
81	E F E F G K P I K I W I G P R V V V F L
301	ACGGATCCCAGAGATGTTGAAGTTATTTTAAGCAGCCACGTGCACATAGACAAATCGCCA
101	T D P R D V E V I L S S H V H I D K S P
361	GAATACAGGCTCTTCGAACCTTGGCTCGGAGATGGTCTGTTGATCAGTACCGGTGAAAAA
121	E Y R L F E P W L G D G L L I S T G E K
421	TGGAGAGCGCACAGAAAACTGATCGCTCCTACTTTCCACTTGAATGTGCTGAAATCATTC
141	W R A H R K L I A P T F H L N V L K S F
481	ATCAGCATTTTCAACAAGAACAGCAGAGCTATTGTAGAAAAAATGAGGAAACTTGGTGAC
161	I S I F N K N S R A I V E K M R K L G D
541	AGAGAATTCGACGTGCACGAATATATGGGAGAAGCGACGGTAGAAATTTTACTCGAAACT
181	R E F D V H E Y M G E A T V E I L L E T
601	GCGATGGGAGTTGATAAGAAAACTCAGGATAGGGAAGCCAGCGAATATGTTATATCAGTC
201	A M G V D K K T Q D R E A S E Y V I S V
661	ATGAAGATGTGCGATATCTTACATTTGAGACATAGACAAATCTGGTTCAGACCTGAAGTC
221	M K M C D I L H L R H R Q I W F R P E V
721	ATTTTCCAGTTCACCAAATACGCTAAAGCTCAAGAAAGATTTTTGAGCATCATTCACGGT
241	I F Q F T K Y A K A Q E R F L S I I H G
781	TTGACCAACAAAGTCCTCCAAGTCAAAAAATCCGAATACGAGAAGAGGAAAAAGGAATTA
261	L T N K V L Q V K K S E Y E K R K K E L
841	CTCGACCATGAACCCGAATTCAACATTCAAGATAAATCGGAAGACTTAAAAACCGAAAAA
281	L D H E P E F N I Q D K S E D L K T E K
901	GCCGAAGTCAAAGTCGAAGAAATCAAATCGAACAAACCCGAAGAAACGGTTATCGGACAC
301	A E V K V E E I K S N K P E E T V I G H
961	TCATACGGCCAAGCAGCTGGATTAAAGGATGATCTCGATGTAGACGATGATATCGGTGAA
321	S Y G Q A A G L K D D L D V D D D I G E
1021	AAGAAACGTCCAGCTTTCTTAGACTTGTTAATTGAAAGCGCCGAAAATGGCGTCGTCTTA
341	K K R P A F L D L L I E S A E N G V V L
1081	ACTGATAAAGAAGTCAGAGAACAAGTCGATACCATCATGTTCGAGGGACACGATACGACA
361	T D K E V R E Q V D T I M F E G H D T T
1141	GCCGCTGGAAGCAGTTTCTTCTTGTGTTTAATGGGTACTCGCCCTGATATCCAGGAAAAA
381	A A G S S F F L C L M G T R P D I Q E K
1201	GTAGTGGAGGAATTGAACACCATTTTCAAAGGCTCTGATCGTCCGTGTACTTTCCAAGAT
401	V V E E L N T I F K G S D R P C T F Q D
1261	ACCTTGGAGATGAAATATTTGGAACGTTGTATTATGGAAACTCTTCGTATGTACCCCCCG
421	T L E M K Y L E R C I M E T L R M Y P P
1321	GTGCCAATGATTGCACGTCAGATCAAAGAGGATATTGTATTACCATCCACCAACGCCATT
441	V P M I A R Q I K E D I V L P S T N A I
1381	GTACCGGCTGGCTGCACCATCGTAATTGGAACGTTCAAGTTACACCGTAACCCAACCGTA
461	V P A G C T I V I G T F K L H R N P T V
1441	TACCCAAATCCGGACGAATACAACCCCGACAACTTCCTACCGGAGAAATCAGCAAACCGT
481	Y P N P D E Y N P D N F L P E K S A N R
1501	CACTACTACGCTTACGTTCCATTCAGCGCTGGACCTCGTAGCTGCGTTGGACGTAAATAC
501	H Y Y A Y V P F S A G P R S C V G R K Y
1561	GCCATGTTGAAATTGAAAATCTTACTTTCCACCATCTTACGTAATTTCCACGTTCGCGCT
521	A M L K L K I L L S T I L R N F H V R A
1621	TCGAAAAACCAAACCGAATGGCAATTACAAGCGGATATCATTTTGAAACGAACTGATGGT
541	S K N Q T E W Q L Q A D I I L K R T D G
1681	TTTAGAATCACTTTGGAGCCGAGAAAGAAGACTAAATTGGTGGCATAA
561	F R I T L E P R K K T K L V A *

K. Nucleotide and deduced amino acid sequences of PsCYP305A22 from *P. solenopsis*

```
1       GACGCAACTTTCACTGCCTATGTTTTAAAGGAATACAAAACACCCACAGTTTCAGTCATG
1        D  A  T  F  T  A  Y  V  L  K  E  Y  K  T  P  T  V  S  V  M
61      TCGCTTTTTATTATTGCTTGCATTTTCATCATCACAGTTTTATATTTGATCTCAACCGTG
21       S  L  F  I  I  A  C  I  F  I  I  T  V  L  Y  L  I  S  T  V
121     ATAAAGCCATCAAATTATCCTCCAGGTCCCAGTCGATTACCTATTGTTGGGAACACGTTT
41       I  K  P  S  N  Y  P  P  G  P  S  R  L  P  I  V  G  N  T  F
181     TTGATCAAGAAATTAAGCAAAGAATTAGGTGGTCAATTCAAAGCTCTTTTGAAGCTGCAT
61       L  I  K  K  L  S  K  E  L  G  G  Q  F  K  A  L  L  K  L  H
241     GAAGATTACAAGTCAAATATTATAGGATTGAAATTAGGCGGTGATCAATATGTTGTGGTT
81       E  D  Y  K  S  N  I  I  G  L  K  L  G  G  D  Q  Y  V  V  V
301     TTTGGACGTAAATTGGTGCAACAGGTGTTTACCAAGGATGAATTCCAAGGCAGACCAGAT
101      F  G  R  K  L  V  Q  Q  V  F  T  K  D  E  F  Q  G  R  P  D
361     GGTTTCTTTATTCGGTTAAGAACCATGGGTACCAAAAAAGGAATTACCATGACTGATGGT
121      G  F  F  I  R  L  R  T  M  G  T  K  K  G  I  T  M  T  D  G
421     AAATTATGGCACGAACAAAGAAATTTCGCTGAAAGACAGCTCAGACAACTAGGTTTCGGC
141      K  L  W  H  E  Q  R  N  F  A  E  R  Q  L  R  Q  L  G  F  G
481     AAATTAGTTACCGAAAATCTCATCAAAGATGAGCTAAACAAATTATTGAATTCTTTCGGA
161      K  L  V  T  E  N  L  I  K  D  E  L  N  K  L  L  N  S  F  G
541     GAAAAACAAGAAGACATTTCGCTTAATCAAAAACTTTCATCAGCCTTCTTGAACGTATTG
181      E  K  Q  E  D  I  S  L  N  Q  K  L  S  S  A  F  L  N  V  L
601     TGGACAATCACTGGGGGCAAGCAGTTCTCGAAAGATGACGATAAATTACCAGCGTTGCTT
201      W  T  I  T  G  G  K  Q  F  S  K  D  D  D  K  L  P  A  L  L
661     AATTTATTACGCGAACGTAGCAAGGCTTTTGATATGGCTGGTGGGTTATTGAATCAACTA
221      N  L  L  R  E  R  S  K  A  F  D  M  A  G  G  L  L  N  Q  L
721     CCGTGGTTGAGATTTATCGCTCCTGATTATTGTGGTTATACCTGTATTCAAAGATTGAAT
241      P  W  L  R  F  I  A  P  D  Y  C  G  Y  T  C  I  Q  R  L  N
781     TCGAAATTATACGAGTTGTTCCAAGAAATCATTGACGAACACAAAAGAACTATTACCAAC
261      S  K  L  Y  E  L  F  Q  E  I  I  D  E  H  K  R  T  I  T  N
841     GAGACTAGGGATTTTGTTGATGCTTATTTACACGAAAAAGAATCTGCTAATCCAGACACC
281      E  T  R  D  F  V  D  A  Y  L  H  E  K  E  S  A  N  P  D  T
901     AGCACATTCACCGATGATCAGCTAGTCGCCATATGTCTAGATTTCTTCATTGCTGGAGCT
301      S  T  F  T  D  D  Q  L  V  A  I  C  L  D  F  F  I  A  G  A
961     CTTACCACTAGTTATACTTTAGATTTTGCAGTCTTAGCCACCGTCAGCCATCCGCATGTG
321      L  T  T  S  Y  T  L  D  F  A  V  L  A  T  V  S  H  P  H  V
1021    CAACAAAAACTTCACGAAGAAATCGACAAAGTATTAAAGCGTAAACAAATGCCATCGATT
341      Q  Q  K  L  H  E  E  I  D  K  V  L  K  R  K  Q  M  P  S  I
1081    GAAGACAAATTCAAATTACCGTATGTCGAAGCGTTGCTCCTTGAATCACAGAGATTTCAA
361      E  D  K  F  K  L  P  Y  V  E  A  L  L  L  E  S  Q  R  F  Q
1141    CACGTTGTACCAACTGCTGGTCCAAGAAGAGTGTTGAAAGATACTACTTTAGATGGATAC
381      H  V  V  P  T  A  G  P  R  R  V  L  K  D  T  T  L  D  G  Y
1201    CGTATTCCAAAGGATACAATCGTTTTAATGAGTTTGAGATCGATTCACTACGACCAGCAA
401      R  I  P  K  D  T  I  V  L  M  S  L  R  S  I  H  Y  D  Q  Q
1261    AGATGGAGTGACCCAGAGGTATTCAGACCAGAAAGATTTCTCACTGAAGATGGACAACTT
421      R  W  S  D  P  E  V  F  R  P  E  R  F  L  T  E  D  G  Q  L
1321    ATCCCTGATGAAGGCTTATGTACATTTGGGTTAGGAAAAAGACGATGCCCTGGTGAAATT
441      I  P  D  E  G  L  C  T  F  G  L  G  K  R  R  C  P  G  E  I
1381    CTAGCGAAAAGTTTCTTATTTATGGCGTTCACCGCGTTATTCAACCATTATAAAATCACA
461      L  A  K  S  F  L  F  M  A  F  T  A  L  F  N  H  Y  K  I  T
1441    TTTCCACCTGGTAAAGAGCCTTCATCTAAAATACCAGCGGCTGGAATATTACTAACGCCT
481      F  P  P  G  K  E  P  S  S  K  I  P  A  A  G  I  L  L  T  P
1501    CAACCTTACTCGGTAAATTTGCAAGCTCGTGGTGATTATGATTTGTGA
501      Q  P  Y  S  V  N  L  Q  A  R  G  D  Y  D  L  *
```
