# Supplementary material for: Expression Patterns, Molecular Characterization, and Response to Host Stress of CYP Genes from Phenacoccus solenopsis (Hemiptera: Pseudococcidae)
Source: Insects. 2019 Aug 22;10(9):264. doi: 10.3390/insects10090264 (PMC6780606; doi:10.3390/insects10090264)
Supplement: Supplementary file 1 [file insects-10-00264-s001.zip › insects-560348-suppl-proof done/supplymentary materials/Table S1-S4.doc]

**Table S1.** Summary of the *Phenacoccus solenopsis* transcriptom.

| **Index** | **All** | **GC%** | **Min Length** | **Median Length** | **Max Length** | **Total Assembled Bases** | **N50** |
| --- | --- | --- | --- | --- | --- | --- | --- |
| Transcript | 48,204 | 37.05 | 201 | 516.00 | 13,825 | 47,635,589 | 1807 |
| Gene | 35,352 | 36.69 | 201 | 403.00 | 13,825 | 30,633,522 | 1680 |

**Table S2.** Annotation results from the statistics of *P. solenopsis.*

| **Gene-Number** | **Swiss-Prot** | **NR** | **Pfam** | **KEGG** | **eggNOG** | **GO** |
| --- | --- | --- | --- | --- | --- | --- |
| 35352.00 | 8980.00 | 12372.00 | 10271.00 | 6883.00 | 11886.00 | 9977.00 |
| 100.00% | 25.4% | 35% | 29.05% | 26.79% | 33.62% | 28.22% |

NR: non-redundant protein database, KEGG: Kyoto Encyclopedia of Genes and Genomes, eggNOG: evolutionary genealogy of genes: Non-supervised Orthologous Groups, Go: Gene Ontology.

**Table S3.** BLASTX best hit results of the CYP genes of *P. solenopsis*.

| **Clan/Gene Name** | **BLASTX Best Hit** | | | |
| --- | --- | --- | --- | --- |
|  | **Gene Name/Species** | **Acc. No.** | **E-Value** | **Identity (%)** |
| CYP2 clan |  |  |  |  |
| *CYP18A1* | Cytochrome P450 18a1[*Frankliniella occidentalis*] | XP_026272510.1 | 0 | 55 |
| *CYP303A1* | PREDICTED: cytochrome P450 303a1[*Acyrthosiphon pisum*] | XP_001951093.1 | 0 | 79 |
| *CYP305A22* | Cytochrome P450 305A13[*Sogatella furcifera*] | AQM57070.1 | 7E-176 | 53 |
| CYP3 clan |  |  |  |  |
| *CYP6PU1* | Probable cytochrome P450 6a13[*Anoplophora glabripennis*] | XP_018571707.1 | 5E-154 | 46 |
| *CYP6PV1* | Putative cytochrome P450 6a14[*Blattella germanica*] | PSN44032.1 | 9E-140 | 44 |
| *CYP6PW1* | Probable cytochrome P450 6a13[*Cryptotermes secundys*] | XP_023711031.1 | 2E-139 | 42 |
| *CYP6PX1* | Putative cytochrome P450 6a14[*Blattella germanica*] | PNF28547.1 | 1E-160 | 49 |
| *CYP6PY1* | Probable cytochrome P450 6a13[*Cryptotermes secundys*] | XP_023711031.1 | 9E-125 | 42 |
| *CYP6PZ1* | Probable cytochrome P450 6a13 isoform X6[*Cryptotermes secundys*] | XP_023709401.1 | 3E-152 | 46 |
| *CYP6PZ2* | Probable cytochrome P450 6a14 isoform X2[*Cryptotermes secundys*] | XP_023709400.1 | 9E-153 | 45 |
| *CYP6PZ3* | Probable cytochrome P450 6a14[*Cryptotermes secundys*] | XP_023705592.1 | 1E-150 | 44 |
| *CYP6PZ4* | Putative cytochrome P450 6a14[*Cryptotermes secundys*] | PNF35811.1 | 2E-156 | 46 |
| *CYP6PZ5* | Putative cytochrome P450 6a14[*Cryptotermes secundys*] | XP_023712679.1 | 1E-139 | 42 |
| *CYP6PZ6* | Probable cytochrome P450 6a14[*Cryptotermes secundys*] | XP_023712679.1 | 6E-132 | 42 |
| *CYP6QA1* | PREDICTED:probable cytochrome P450[*Bemisia tabaci*] | XP_018901643.1 | 4E-148 | 44 |
| *CYP6QB1* | Probable cytochrome P450 6a14 isoform X2[*Cryptotermes secundys*] | XP_023705445.1 | 7E-150 | 43 |
| *CYP6QD1* | Putative cytochrome P450 6a14[*Blattella germanica*] | PSN44032.1 | 6E-61 | 36 |
| CYP4 clan |  |  |  |  |
| *CYP4G219* | PREDICTED: probable cytochrome P450 4g15-like isoform X1[*Bemisia tabaci*] | XP_018915012.1 | 0 | 69 |
| *CYP4NT1* | Cytochrome P450 4CK1[*Aphis gossypii*] | ATN96011.1 | 2E-168 | 51 |
| *CYP4NU1* | Cytochrome P450 4C1-like[*Onthophagus taurus*] | XP_021934353.1 | 5E-124 | 42 |
| *CYP4NV1* | Cytochrome P450 4C1[*Cryptotermes secundys*] | PNF27071.1 | 1E-121 | 43 |
| *CYP4NV2* | Cytochrome P450 4DC1[*Nilaparvata lugens*] | AIW80006.1 | 2E-121 | 43 |
| *CYP380C41* | Cytochrome P450 4C1-like[*Rhopalosiphum maidis*] | XP_026815184.1 | 0 | 57 |
| *CYP380H1* | Cytochrome P450 4C1-like[*Melanaphis sacchari*] | XP_025197496.1 | 1E-153 | 44 |
| Mitochondrial clan |  |  |  |  |
| *CYP301A1* | PREDICTED: probable cytochrome P450 301a1,mitochodrial-like isoform X1[*Nicrophorus vespilloides*] | XP_017784363.1 | 0 | 75 |
| *CYP301B1* | Probable cytochrome P450 49a1[*Rhopalosiphum maidis*] | XP_026814525.1 | 0 | 63 |
| *CYP302A1* | Cytochrome P450 302a1,mitochodrial-like isoform X2[*Melanaphis sacchari*] | XP_025209121.1 | 0 | 59 |
| *CYP315A1* | Cytochrome P450 315a1,mitochodrial[*Myzus persicae*] | XP_022164033.1 | 2E-129 | 44 |
| *CYP353F1* | Probable cytochrome P450 49a1[*Anoplophora glabripennis*] | XP_018577263.1 | 4E-128 | 45 |
| New Clan |  |  |  |  |
| *CYP3633A1* | PREDICTED: probable cytochrome P450 6a13[*Diuraphis noxia*] | XP_015371246.1 | 2E-97 | 38 |
| *CYP3633A2* | Cytochrome P450 6a13-like[*Myzus persicae*] | NP_001352549.1 | 6E-100 | 37 |
| *CYP3634A1* | Cytochrome P450 4C1-like[*Myzus persicae*] | XP_022175937.1 | 4E-115 | 38 |
| *CYP3635A2* | Cytochrome P450 monooxygenase CYP6BR4[*Tenebrio molitor*] | AKE17696.1 | 2E-95 | 36 |
| *CYP3636A1* | Cytochrome P450 6a2[*Bombus terrestris*] |  | 7E-110 | 36 |
| *CYP3638A1* | Cytochrome P450 4C1-like isoform X1[*Myzus persicae*] | XP_022166733.1 | 2E-77 | 33 |
| *CYP3638A2* | PREDICTED: cytochrome P450 4C1[*Acyrthosiphon pisum*] | XP_008179606.2 | 3E-83 | 33 |
| *CYP3638B1* | Cytochrome P450 4C1-like [*Myzus persicae*] | XP_022175937.1 | 3E-94 | 35 |

**Table S4.** *CYP* gene family proteins used for the phylogenetic analysis deduced amino acid sequences with homologues from other species of insects deposited in the NCBI data.

| **Gene** | **Species** | **Number of**  **Amino Acids** | **GenBank number** | **Identity**  **(%)** | **E Value** |
| --- | --- | --- | --- | --- | --- |
| EmeCYP18a1 | *Eufriesea mexicana* | 539 | XP_017764635.1 | 51 | 0.0 |
| BteCYP18a1 | *Bombus terrestris* | 539 | XP_003400174.1 | 53 | 0.0 |
| MroCYP18a1 | *Megachile rotundata* | 538 | NP_001351746.1 | 53 | 0.0 |
| NleCYP18a1 | *Neodiprion lecontei* | 537 | XP_015516782.1 | 54 | 0.0 |
| ZneCYP18a1 | *Zootermopsis nevadensis* | 541 | XP_021914922.1 | 54 | 0.0 |
| FocCYP18a1 | *Frankliniella occidentalis* | 542 | XP_026272510.1 | 54 | 0.0 |
| DnoCYP303a1 | *Diuraphis noxia* | 499 | XP_015370810.1 | 79 | 0.0 |
| ApiCYP303a1 | *Acyrthosiphon pisum* | 500 | XP_001951093.1 | 79 | 0.0 |
| MpeCYP303a1 | *Myzus persicae* | 499 | XP_022166722.1 | 79 | 0.0 |
| MsaCYP303a1 | *Melanaphis sacchari* | 499 | XP_025204279.1 | 78 | 0.0 |
| AgoCYP303a1 | *Aphis gossypii* | 499 | ATN95982.1 | 79 | 0.0 |
| RmaCYP303a1 | *Rhopalosiphum maidis* | 499 | XP_026808848.1 | 79 | 0.0 |
| SfuCYP305A13 | *Sogatella furcifera* | 475 | AQM57070.1 | 53 | 7E-176 |
| LstCYP305A13v2 | *Laodelphax striatella* | 476 | AFU86441.1 | 52 | 1E-173 |
| CseCYP305a1 | *Cryptotermes secundus* | 503 | PNF29828.1 | 52 | 2E-171 |
| NluCYP305A15 | *Nilaparvata lugens* | 494 | AIW79974.1 | 51 | 5E-171 |
| NluCYP305a1 | *Nilaparvata lugens* | 507 | XP_022195997.1 | 51 | 1E-168 |
| AgoCYP305A1 | *Aphis gossypii* | 488 | ATB55046.1 | 48 | 3E-166 |
| BtaCYP4g15 | *Bemisia tabaci* | 583 | XP_018915012.1 | 69 | 0.0 |
| AglCYP4G51 | *Aphis glycines* | 566 | AXJ21604.1 | 68 | 0.0 |
| AgoCYP4G51 | *Aphis gossypii* | 566 | ATN95994.1 | 68 | 0.0 |
| SflCYP4g15 | *Sipha flava* | 566 | XP_025404746.1 | 68 | 0.0 |
| BtaCYP4G68 | *Bemisia tabaci* | 587 | AYN79709.1 | 69 | 0.0 |
| DnoCYP4g15 | *Diuraphis noxia* | 566 | XP_015373315.1 | 68 | 0.0 |
| AaeCYP4d1 | *Aedes aegypti* | 505 | XP_021705200.1 | 39 | 8E-120 |
| OtaCYP4C1 | *Onthophagus taurus* | 500 | XP_022915869.1 | 41 | 1E-113 |
| ZneCYP4C1-X1 | *Zootermopsis nevadensis* | 496 | XP_021934353.1 | 42 | 5E-124 |
| RflCYP4U3v1 | *Reticulitermes flavipes* | 508 | ABB86762.2 | 42 | 6E-121 |
| NluCYP4DC1 | *Nilaparvata lugens* | 502 | AIW80006.1 | 43 | 2E-123 |
| NfuCYP4DC1 | *Sogatella furcifera* | 505 | AQM57056.1 | 42 | 1E-120 |
| CseCYP4C1 | *Cryptotermes secundus* | 480 | XP_023705844.1 | 42 | 9E-117 |
| RmaCYP4C1 | *Rhopalosiphum maidis* | 508 | XP_026815628.1 | 48 | 8E-173 |
| AgoCYP4CK1 | *Aphis gossypii* | 508 | ATN96011.1 | 51 | 2E-168 |
| MsaCYP4C1 | *Melanaphis sacchari* | 538 | XP_025197496.1 | 44 | 1E-153 |
| MpeCYP4C1 | *Myzus persicae* | 543 | XP_022175934.1 | 44 | 7E-153 |
| ApiCYP4C1 | *Acyrthosiphon pisum* | 545 | XP_001951034.3 | 43 | 6E-151 |
| AgoCYP380C6 | *Aphis gossypii* | 546 | ATN95989.1 | 55 | 0.0 |
| SflCYP4C1 | *Sipha flava* | 536 | XP_025418745.1 | 33 | 6E-93 |
| MpeCYP6a13 | *Myzus persicae* | 508 | NP_001352549.1 | 37 | 6E-100 |
| ApiCYP6a14 | *Acyrthosiphon pisum* | 518 | XP_001945100.2 | 35 | 1E-97 |
| MpeCYP6CY3 | *Myzus persicae* | 511 | AHB52749.1 | 35 | 2E-95 |
| AglCYP6CY20 | *Aphis glycines* | 509 | AXJ21603.1 | 36 | 8E-98 |
| DnoCYP6a13 | *Diuraphis noxia* | 513 | XP_015371246.1 | 38 | 2E-97 |
| RmaCYP6a13 | *Rhopalosiphum maidis* | 511 | XP_026810291.1 | 37 | 2E-95 |
| CfeCYP6a14 | *Ctenocephalides felis* | 510 | XP_026478207.1 | 35 | 8E-100 |
| TmoCYP6BQ37 | *Tenebrio molitor* | 519 | AKZ17696.1 | 35 | 3E-98 |
| ZneCYP6a13 | *Zootermopsis nevadensis* | 507 | XP_021919178.1 | 37 | 2E-97 |
| OtaCYP6a20 | *Onthophagus taurus* | 494 | XP_022910877.1 | 34 | 3E-96 |
| TmoCYP6BR4 | *Tenebrio molitor* | 498 | AKZ17697.1 | 36 | 2E-95 |
| BteCYP6a2 | *Bombus terrestris* | 500 | XP_003400131.1 | 36 | 7E-110 |
| TcaCYP6BK6 | *Tribolium castaneum* | 529 | KYB24704.1 | 35 | 2E-107 |
| LliCYP6X1v1 | *Lygus lineolaris* | 516 | AAL15173.1 | 42 | 1E-131 |
| ZneCYP6k1 | *Zootermopsis nevadensis* | 523 | XP_021934435.1 | 44 | 2E-141 |
| TmoCYP6BQ34 | *Tenebrio molitor* | 520 | AKZ17693.1 | 42 | 5E-144 |
| AalCYP6a14 | *Aedes albopictus* | 498 | XP_019538127.1 | 42 | 9E-144 |
| CseCYP6a14 | *Cryptotermes secundus* | 529 | XP_023712679.1 | 42 | 6E-132 |
| CseCYP6k1 | *Cryptotermes secundus* | 524 | PNF15722.1 | 40 | 3E-128 |
| CseCYP6a14 | *Zootermopsis nevadensis* | 531 | XP_021934426.1 | 40 | 2E-125 |
| CseCYP6a13 | *Cryptotermes secundus* | 525 | XP_023705592.1 | 43 | 1E-153 |
| TcaCYP6a14 | *Tribolium castaneum* | 524 | PNF15722.1 | 44 | 8E-151 |
| AglCYP6a13 | *Anoplophora glabripennis* | 506 | XP_018571707.1 | 46 | 5E-154 |
| TcaCYP6a2 | *Tribolium castaneum* | 514 | XP_974742.1 | 43 | 1E-153 |
| DheCYP6QB22 | *Dastarcus helophoroides* | 515 | AGJ51945.1 | 42 | 6E-153 |
| BgeCYP6a14 | *Blattella germanica* | 511 | PSN44032.1 | 44 | 9E-140 |
| BtaCYP6a13 | *Bemisia tabaci* | 504 | XP_018901643.1 | 44 | 2E-148 |
| NluCYP6AX1 | *Nilaparvata lugens* | 514 | CAH65681.2 | 43 | 4E-144 |
| CYP6AX1v2 | *Nilaparvata lugens* | 514 | AIW79980.1 | 43 | 2E-143 |
| CYP6AX3 | *Sogatella furcifera* | 503 | AQM57043.1 | 43 | 8E-142 |
| AtuCYP12A2 | *Aethina tumida* | 469 | XP_019871673.1 | 44 | 6E-130 |
| TcaCYP12A2 | *Tribolium castaneum* | 494 | XP_008190935.1 | 42 | 6E-128 |
| AgoCYP49a1 | *Anoplophora glabripennis* | 470 | XP_018577263.1 | 45 | 4E-128 |
| SflCYP49a1 | *Sipha flava* | 452 | XP_025425319.1 | 43 | 2E-127 |
| RmaCYP49a1 | *Rhopalosiphum maidis* | 451 | XP_026816007.1 | 44 | 2E-126 |
| TcaCYP353A1 | *Tribolium castaneum* | 478 | EFA01331.2 | 42 | 2E-128 |
| MpeCYP315a1 | *Myzus persicae* | 477 | XP_022164033.1 | 44 | 2E-129 |
| ZneCYP315a1 | *Zootermopsis nevadensis* | 465 | XP_021924799.1 | 41 | 3E-129 |
| MsaCYP315a1 | *Melanaphis sacchari* | 476 | XP_025195090.1 | 42 | 3E-125 |
| DnoCYP315a1 | *Diuraphis noxia* | 477 | XP_015380369.1 | 42 | 4E-125 |
| ApiCYP315a1 | *Acyrthosiphon pisum* | 477 | XP_001944183.2 | 42 | 2E-124 |
| RmaCYP315a1 | *Rhopalosiphum maidis* | 459 | XP_026818325.1 | 42 | 3E-124 |
| BtaCYP49a1 | *Bemisia tabaci* | 525 | XP_018918048.1 | 60 | 0.0 |
| RmaCYP49a1 | *Rhopalosiphum maidis* | 517 | XP_026814525.1 | 63 | 0.0 |
| ApiCYP49a1 | *Acyrthosiphon pisum* | 517 | XP_001946744.2 | 63 | 0.0 |
| MsaCYP49a1 | *Melanaphis sacchari* | 528 | XP_025204934.1 | 63 | 0.0 |
| DnoCYP49a1 | *Diuraphis noxia* | 539 | XP_015363191.1 | 62 | 0.0 |
| AgoCYP49A1 | *Aphis gossypii* | 517 | ATN96003.1 | 62 | 0.0 |
| NveCYP301a1 | *Nicrophorus vespilloides* | 537 | XP_017784363.1 | 75 | 0.0 |
| AgoCYP301A1 | *Aphis gossypii* | 525 | ATN96002.1 | 70 | 0.0 |
| BtaCYP301a1 | *Bemisia tabaci* | 542 | XP_018916684.1 | 73 | 0.0 |
